# Barriers and Facilitators to implementation of the Free Water Protocol in the Acute Stroke Unit Setting: A Mixed Methods Systematic Review

**DOI:** 10.1007/s00455-025-10805-7

**Published:** 2025-02-05

**Authors:** Sabrina A. Eltringham, Nicola Martindale, Elizabeth Lightbody, Sue Pownall, Andrew Booth, Craig J. Smith

**Affiliations:** 1 Sheffield Teaching Hospitals NHS Foundation Trust, Sheffield, S10 2JF UK; 2 Sheffield Hallam University, Sheffield, S1 1WB UK; 3 University of Central Lancashire, Preston, PR1 2HE UK; 4 Sheffield Centre for Health and Related Research (SCHARR), University of Sheffield, Sheffield, S1 4DA UK; 5 Division of Cardiovascular Sciences, University of Manchester, Manchester, M13 9PT UK; 6 Manchester Centre for Clinical Neurosciences, Geoffrey Jefferson Brain Research Centre, Salford Royal Hospital, Northern Care Alliance NHS Trust, Manchester, M6 8HD UK

**Keywords:** Deglutition, Deglutition disorders, Dysphagia, Acute stroke, Free water protocol

## Abstract

Free water protocols (FWP) give patients at risk of aspiration the option to drink water between meals. Evidence is lacking about their use in acute stroke care. This systematic review evaluated the literature about barriers and facilitators to FWP implementation in acute stroke unit settings. Electronic databases and grey literature sources were systematically searched, eligible studies were critically appraised, and data extracted and mapped onto the Consolidated Framework for Implementation Research (CFIR). The results are presented in a narrative synthesis. Five studies were identified for inclusion: Two qualitative studies, 1 mixed method study, 1 randomised controlled trial and 1 pilot cohort study. Barriers and facilitators to implementation were identified across the 5 CFIR domains. Key barriers were a lack of evidence base and a standard protocol, trying to adapt and deliver a protocol designed for a different setting, complexity of patient selection and FWP design, culture of risk aversion, nursing staff availability and skills to deliver the FWP, and a greater use of agency nurses and transient workforce. Key facilitators were the existence of national guidance for research into its use, implementation of oral care protocols prior to FWP implementation, the unique characteristics of the acute stroke setting, leadership and modelling by senior clinicians, interdisciplinary working and accountability for roles and responsibilities for each discipline, regular communication and ongoing education, and involving patients in decision making and implementation. The findings of this review will guide the data collection of a feasibility study of the FWP in acute stroke.

**Supplementary Information:**

The online version contains supplementary material available at 10.1007/s00455-025-10805-7.

## Introduction

Dysphagia affects more than 50% of acute stroke patients, increases risk of stroke-associated pneumonia, malnutrition, and dehydration [1], and reduces quality of life [2]. Treatment of post stroke dysphagia involves compensation and rehabilitation approaches to improve the safety and efficiency of the swallow. Patients are often recommended to drink thickened fluids as they move more slowly in the mouth and pharynx [3], maintain cohesiveness [4], and increase the speed and extent of laryngeal movements [5], thereby increasing the safety of the swallow [6] and reducing risk of pneumonia [7]. However, many people dislike thickened fluids. This can lead to reduced fluid intake and dehydration [8] which increases the risk of complications such as extension of the presenting stroke or recurrent strokes [9, 10], venous thromboembolism [11] and increased mortality [12].

The free water protocol (FWP) [13] gives patients who are designated nothing by mouth (NBM) or prescribed thickened fluids the option to drink water on the basis that if aspirated in small amounts, the inert pH characteristics of water will not harm the lungs as it is absorbed into the bloodstream via aquaporins [14]. Guidelines have been produced to minimise the risk of adverse consequences of aspiration, including regular oral hygiene, water only between meals, and patient specific precautions. A meta-analysis of the data from rehabilitation studies, which included stroke patients, found no significant increase in lung complications when following a FWP, fluid intake may increase together with a trend for improved quality of life (QOL) [15]. There is also a suggestion that giving patients an option to ‘practice’ drinking thin fluids in the relatively safe form of water may help to maintain and improve the swallowing muscular system (‘rehabilitate the swallow’) [16].

The stroke pathway has clearly defined stages. People with suspected stroke are directly admitted to a hyperacute stroke unit where they typically spend the first 72 h undergoing rapid assessment and treatment. Most stroke patients need further hospital care and will be moved to an acute stroke unit where they receive input for medical and neurological complications of their stroke and early rehabilitation [17]. The acute phase of care is usually considered to have ended at the time of discharge from the acute stroke unit or 30 days after hospital admission [18]. Once patients are medically ready to leave the acute hospital setting they may be transferred to a rehabilitation unit or discharged home with community support.

Little evidence exists for the use of the FWP in acute stroke unit settings. Studies have encountered challenges in recruiting participants [16, 19]. Murray et al. [20] hypothesised this difficulty is related to the acuity and complexity of patient presentations in acute care and the exclusion criteria, or the nature of the acute setting itself rendering the FWP implementation unfeasible. This systematic review aimed to answer the question “What are the barriers and facilitators to implementation of the Free Water Protocol in acute stroke unit settings?” The review's objective was to establish factors affecting implementation to inform a feasibility study of implementation of the FWP in the acute stroke unit setting.

## Methods

The protocol for the systematic review was registered prospectively on PROSPERO (Registration number CRD42023470349) and has been reported in accordance with the Preferred Reporting Items for Systematic Reviews and Meta-Analyses (PRISMA) guidelines [21]. The research question: What are the barriers and enablers to implementation of the Free Water Protocol in acute stroke unit settings?, was formulated using the population-intervention-comparison-outcome (PICO) framework [22] which determined the inclusion and exclusion criteria for study selection (Supplementary Material—Table 1). The population (P) was acute stroke patients with dysphagia, the intervention (I) was the FWP, the comparison (C) was usual stroke care, and the outcome (O) was establishing factors affecting implementation of the FWP in acute stroke unit settings. For this review the term ‘barriers’ were defined as factors that hindered the successful implementation of the FWP in the acute stroke unit setting and ‘facilitators’ were factors that promoted implementation. Peer reviewed published studies, conference proceedings and abstracts of the FWP intervention in stroke patients in the hospital acute stroke units were eligible for inclusion. If the setting was not explicitly stated as a hospital acute stroke unit, outcome measures were reviewed for temporal indicators. There was no restriction on language or study design. Non stroke, or mixed population studies where information about stroke patients could not be extracted, and studies undertaken in non-acute settings were excluded. A phrase-based Google Scholar search was conducted and scanned for alternative terms for the ‘Free Water Protocol’ to formulate the free text search strategy (Supplementary Material—Table 2). A phrase-based search was positively indicated given (i) the precision of the phrase in capturing the intervention of interest; (ii) the lack of alternative synonyms for conveying the topic; (iii) the non-availability of index terms relating to the focus of interest and (iv) the fact that: “free”, “water” and protocol” are all very common single words and “free water” combined with “protocol” and “water protocol” combined with free would not yield additional records. In constructing the search the implications of not using the Boolean AND were explored with no additional references being identified within the test set. Electronic databases: CINAHL (EBSCOhost), MEDLINE (Ovid), EMBASE (Ovid) and Cochrane Library (Wiley) were searched from inception to 11/10/2023. Grey literature sources (Google Scholar and domain-based Google searches) were searched for reports, dissertations, theses, and conference abstracts. Reference lists and citation searches of selected studies were scanned for relevant studies. Covidence software was used to screen the results. Two reviewers (SAE, NM) independently piloted the inclusion/exclusion criteria on 10% of the titles/abstracts of the retrieved studies before independently assessing the remaining titles/abstracts for eligibility for inclusion. The inclusion criteria were reapplied independently to the full texts of the remaining articles by the same two reviewers. Any differences between judgements were resolved through discussion between the two reviewers at each stage. Reasons for excluding studies at full text stage were recorded for transparency.

**Table 1:** Study characteristics

Study	Design, setting, country	Participant characteristics	Approach to analysis	Key findings affecting implementation
Barker et al., 2019	Qualitative – Semi structured interviews, 3 tertiary hospitals with dedicated acute stroke services, Australia	26 clinicians (9 nurses, 8 SLPs, 5 doctors and 4 dietitians). Median years of experience 13.5 years and 5 years specifically working with the acute stroke population	Themes mapped to Theoretical Domains Framework (Michie et al., 2005)	*Key facilitators*: patient benefit, existing communication and education systems, unique benefits of the acute hospital and peer support and modelling vs. *Key barriers*: nurses lack of oral care skills, agency nurses lack of stroke specific skills, only SLPs perceived to be involved with FWPs, FWP rules may not get followed and lead to adverse outcomes, FWP increase nursing workload, transient work force and established culture of using thickened fluids
Murray et al. 2022a	Qualitative – Semi structured interviews, 3 tertiary hospitals, Australia	36 clinicians (4 dietitians, 7 medical officers, 8 registered nurses and 17 SLPs), 6 > months experience in an acute stroke or general medicine unit	Themes mapped to Situated Clinical Decision-Making Framework (Gillespie and Peterson, 2009)	*Key facilitator*: Patient preferences (Knowing the Person); workplace culture and modelling of the FWP decision making and implementation by senior colleagues vs. *Key barriers:* Pattern of illness scripts (Knowing the Case), poor functional status (Knowing the Patient), risk averse approach particularly by junior staff and lack of formalised guidelines and protocols
Murray et al., 2022b	Mixed methods—parallel case study design using medical records and semi structured interviews, 2 Acute Hospitals, Australia	3 patient-nurse- SLP triads (included 1 stroke ward stroke patient (P3), nurse-SLP triad)	Data descriptively analysed and triangulated	*Key facilitators:* Clear verbal communication, stroke ward environment vs. *Key barriers* reliance on others, unclear/inaccurate documentation and insufficient documentation, no standard procedures
Kenedi et al., 2019	Quantitative, RCT, Urban Acute Care Hospital with Level 1 trauma and primary stroke centre, United States of America	104 patients (Control group with no access to thin liquids, including water or ice chips N = 52 which inc, N = 17 Stroke patients/Experimental group with access to water and ice chips N = 52 inc. 14 Stroke patients)	Statistical analysis	*Key facilitators*: Non inferior outcomes/no significant differences between groups, established oral care programme vs. *Key barriers*; short stays in acute setting impacting of monitoring outcomes, limited resources for documentation
Weber 2009	Quantitative, Pilot study, Acute Care Stroke Unit, United States of America	5 out of 20 acute patients recruited in 7 months (study in progress at time of publication)	Narrative description	*Key facilitators:* Improved patient quality of life, no medical compromise vs. *Key barriers*: Length of stay in acute care stroke unit, difficulty training staff, staff workloads, SLP reluctance to refer due to the time to set up

*SLP* Speech and language pathologist, *WP* water protocols, *FWP* free water protocol.

**Table 2:** Summary of the barriers (B) and facilitators (F) mapped to the CFIR domains

I. Innovation	
A. Innovation source	Origin of the Frazier Free Water Protocol (F)
B. Innovation evidence-base	Lack of evidence base in the acute stroke setting (B), Lack of clinical guidelines (B)
C. Innovation relative advantage	Non inferior outcomes (F), Positive feedback from patients (F)
D. Innovation adaptability	Patient selection criteria (F), Countering the intent of the FWP design (B)
E. Innovation trialability	Exclusion criteria (B), Short patient stay (B)
F. Innovation complexity	Patient selection (B), Patient specific design (B)
G. Innovation design	Instructions for deliverers (F), Types of materials bundled with the innovation (F)

II. Outer setting	
C. Local conditions	Government enforced changes to the health system (B), Hospital admitting more patients than wards staffed to manage (B)
E. Policies & laws	National guidelines recommendations for research (F)

III. Inner setting	
A. Structural characteristics (work infrastructure)	Regular monitoring of patients (F), Time intensity of acute stroke care (B), Nurses existing heavy workload (B), Transient workforce (B)
B. Relational connections	Teamness and cohesion of the acute stroke ward (F)
C. Communications	Established systems to educate nursing staff (F), Processes to disseminate new protocols (F)
D. Culture (deliverer and learning centeredness)	Learning culture on the acute stroke unit (F), Deliverer beliefs and attitude to risk (B), Routinely providing thickened fluids (B)
F. Compatibility	Basic nursing care (F), Fast pace (B), High turnover of patients (B), Nurses availability (B), Delegation of tasks for dependent patients (B), Limited resources precluded documentation of fluid intake (B)
G. Relative priority	Prioritisation of other duties over the FWP (B)
K. Access to knowledge & information	Patient, family and caregiver education (F), In house training to nurses and physicians (F), Peer support and modelling from supervisors and peers (F), Ongoing staff education (B), No standard procedure/protocol

IV. Individuals characteristics	
A. Need	Perceived benefits to QOL (comfort, normalisation and preferences for care) (F), perceived negative outcomes (aspiration and development of chest complications) (B), patient preferences being outweighed (B)
B. Capability	Staff with high degree of dysphagia expertise (F), Knowledge of the FWP intervention (F), Stroke specific nursing skills (F), Experience of the FWP (F/B), Lack of awareness of the FWP (B), Lack of clarity of instructions (B), Incomplete or unclear documentation (B), Lack of stroke specific skills (new nurses, student or agency nurses (B), Nurses forgetting to get water (B), Measures to mitigate risk not being completed or documented (B), Pattern of illness scripts (B)
C. Opportunity	Extension of current role (F), availability of family support (F), Lack of nursing availability to deliver mouthcare (B), Time and number of staff needed to position patients (B), Lack of nursing availability to supervise patients (B)
D. Motivation	Staff receptivity (F), Patient desire (F), Patient feedback (F), Concerns about legal liability (B), Staff negative attitudes about delivering oral care (B), SLPs reluctance to refer (B), Patient misgivings about discomfort and risk of pneumonia

V. Implementation process	
A. Teaming	MDT communication and collaboration (F), Team support (F), Mindset of FWPs as sole domain of SLPs (B)
B. Assessing needs	Barker et al., Murray et al. 2022a and Murray 2022b conducted semi structured interviews ± information from medical records to collect information about perceptions and experiences (barriers and facilitators) of implementing the decision-making process. Weber’s methods to gather recipient feedback were unclear. Kenedi et al. did not collect information about the priorities, preferences and needs of the individuals involved
C. Assessing context	None of the studies used the CFIR. Other frameworks used included TDF and Situated Clinical Decision-Making Framework
E. Tailoring strategies	Bedside oral care and water tracking sheet for staff /caregivers to complete (F), Communication and collaboration among nurses, physicians, SLPs to monitor participant status (F), Family education to implement the FWP (F) Implementation of oral care protocols before implementing the FWP (F), Daily patient monitoring for signs and symptoms of aspiration (F)
F. Engaging (innovation recipients)	Letting the patient know (F)
G. Doing	Kenedi et al. 3-phase implementation design of the FWP as part of their RCT to evaluate clinical outcomes of the FWP. Weber conducted a pilot study which included implementation outcomes acceptability and fidelity
H. Reflecting & evaluating (implementation and innovation)	Implementation: Combining use of champions and interdisciplinary approach (F), Clearing defined roles and responsibilities (F), Regular communication between clinicians (particularly at handover) (F), Considerable education on FWP (rules, risks, benefits) (F), Strategies to minimise nurses workload (offer water in lieu of thickened fluids, support staff and family members to offer water and supervise patients)(F), Implement oral care protocols before FWPs are implemented (F), formalised guidelines and protocols (F), Leadership by senior clinicians (F), Modelling of decision making and implementation by senior staff (F), Involving patients in the decision making (F), Uncertainty about whether the FWP would be implemented as intended (B) Innovation: Diagnosis based exclusion criteria (B)
I. Adapting	Adaptations made at multiple levels

*FWP* Free water protocol, *QOL* quality of life, *SLPs* speech and language pathologists, *MDT* multidisciplinary team, *CFIR* consolidated framework for implementation research, *TDF* theoretical domains framework, *RCT* randomised control trial

Authors SAE and NM independently conducted a quality assessment of the selected studies using the Critical Appraisal Skills Programme (CASP) Randomised Controlled Trial and Cohort Study Checklists [23] and Mixed Methods Appraisal Tool (MMAT) [24] (Supplementary Material—Table 3). A data extraction form was created to extract key information including study focus and methods, and factors affecting implementation (barriers and facilitators) based on the domains of the Consolidated Framework for Implementation Research (CFIR) [25]. The data extraction form was piloted on two eligible studies to ensure all the relevant information was captured. The first author extracted and mapped the data onto the CFIR domains and NM checked the extracted data. Disagreements between reviewers were resolved through discussion. Data was then summarised in a table with illustrative verbatim text extracts from the included studies. The updated CFIR interview coding guidelines [26] were used to ensure the fidelity with which data was mapped onto the appropriate domain. A third member of the research team (EL) checked the data mapping for trustworthiness. The findings were synthesised qualitatively within a narrative synthesis.

## Results and Discussion

**Fig. 1:**
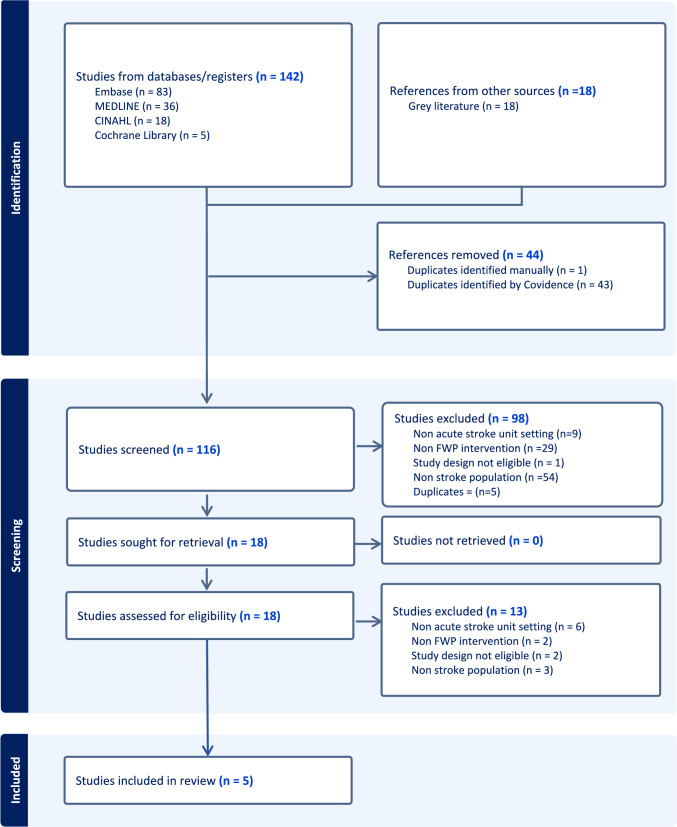
Search methodology and outcome

Searching the Embase, MEDLINE, CINAHL and Cochrane Library databases yielded 142 references and a further 18 references were identified through grey literature sources (Fig. 1—Search methodology and outcome). Of these eighteen full text articles were assessed for eligibility and 5 studies were included (Table 1—Study characteristics). The studies had diverse study designs, including qualitative methods [20, 27], mixed methods [28], a randomised control trial (RCT) [29] and a pilot cohort study [30], making direct comparisons challenging. The heterogeneity of study designs and small number of studies meant a quantitative meta-analysis was not possible. Three of the studies were generated from the same research laboratory [20, 27, 28] which may introduce bias into the overall findings. Clear rationales were provided by the researchers for the choice of methods for the qualitative and mixed methods studies [20, 27, 28]. The results from the different methods used in the mixed methods study [28] were integrated and differences and similarities between the data sources were discussed. Both qualitative studies used theoretical frameworks to help interpret and map their data. One limitation of the mixed methods study [28] was the small number of patient–nurse–SLP triads which included only one acute stroke patient triad which restricts the generalisability of findings. A potential limitation of the qualitative studies was lack of reflexivity from the research team regarding their role and the consequent potential for bias. Several methodological limitations were identified in the pilot study [30]. Limitations included a lack of information about how outcomes were being measured and minimisation of bias, participant characteristics were not discussed meaning confounding factors were unable to be identified, follow up of participants was not complete, and the author was unable to answer the research question due to recruitment challenges. The primary limitations of the RCT study were the low statistical power and small effect sizes, the challenges of blinding research staff, and the failure to split all the findings into stroke and non-stroke populations within the experimental and non-experimental group due to the small sample size.

The findings are presented in alignment with the identified CFIR domains, with quotations from the included studies. The word limit precludes including all the supporting information which is available in the Supplementary Material (Table 4). A summary of the identified barriers and facilitators mapped onto the CFIR are presented in Table 2.

### Domain I: Innovation

All included studies cited the Frazier Free Water Protocol developed by the Frazier Rehabilitation Institute [13] as the origin of the innovation. Lack of evidence and clinical guidelines for use of the FWP in the acute stroke setting was perceived as a barrier to implementation and reinforced the avoidance of its use [20, 27, 28]. Incorporating the FWP into hospital policies and procedures was felt unlikely to happen until there was more empirical evidence on the safety and use in the acute stroke population [27]. Acknowledging limitations for statistical power, in one study no significant group differences were reported for eligible stroke and trauma patients in positive outcomes, and negative clinical indicators did not differ significantly between the control and experimental groups [29]. None of the patients in the pilot study [30] had any medical compromise and recipients reported their quality of life improved. Short length of stay made it challenging to follow up patients for a sufficient length of time to enable measurement of negative and positive outcomes [29, 30].

A complex interaction of factors affecting implementation success were identified [28]. These factors included patient selection and protocol design. Facilitative factors included guidelines to minimise risk of adverse consequences and maximise patient safety [20, 27]. As part of the implementation design one study incorporated the Aspiration Precaution Oral Care program and used in service and written material to educate nursing staff on the study purpose and implementation [29], whilst others included a tracking sheet for oral care and water intake as part of their innovation bundle [30]. Conversely speech and language pathologists (SLPs) were found to design the FWP to be as safe as possible by giving teaspoons of water and if there was no evidence of aspiration following instrumental assessment. Although the FWP can be adapted according to the environment and specific patient condition and needs [13], these adaptations were felt to counter the original intention of the FWP and made it “impossible to assess safety and efficacy outcomes” (P.119) [28]. One study tailored the selection criteria and excluded patients with brain stem strokes [30], referred to as diagnosis-based exclusion criteria [20], whilst others adapted the FWP design by including ice chips [29].

Exclusion criteria were given as reasons for difficulty recruiting to trials [20]. Poor functional status (mobility, cognition or respiration) and SLP red flags (significant oral, swallowing and secretion issues) warranted exclusion by many participants. Risk factors such as levels of alertness, impulsivity, delirium and fatigue were considered to potentially increase risk. Nurses felt that patients on fluid restrictions would be unsuitable. In contrast dietitians felt the amount of water consumed was unlikely to affect a fluid restriction, and that changes could be made around the patient’s non oral feeding to avoid compromising the fluid restriction. Moving forward Murray et al. [20] propose consideration of broader patient function selection criteria alongside modifiable context specific factors.

### Domain II: Outer Setting

Barriers included local conditions such as changes to the health system and the hospital admitting more patients than the ward could manage resulting in greater utilisation of agency and bank nurses who lacked familiarity with the acute stroke environment [27]. National guidelines and recommendation by the National Health Institute for Health Care Excellence (NICE) for research of the benefits of the FWP versus NBM or thickened fluids was identified as a way forward for research trials to be conducted in the acute stroke setting [20]. The updated NICE guidance ‘Stroke rehabilitation in adults’ [31] makes a recommendation for research to investigate the use of the FWP, particularly in studies with a larger number of participants. This is an opportunity to reassess the exclusion criteria and address the evidence gap.

### Domain III: Inner Setting

The cohesiveness of the stroke ward and the frequent contact between nursing staff and patients and regular monitoring were positive characteristics of the acute stroke unit setting [27, 28]: *"Generally, I feel we do reasonably well here cause obviously we are the stroke ward.” N3, P.116 *[28]*.* The heavy workload and time intensity of acute care were identified as challenges for SLPs to educate clinicians and for implementation. This would result in a potential lack of adherence to the FWP guidelines or not implementing the FWP at all [27]: *" My concern would be more about when do you actually fit it in, that you can go on with the workload that you’ve already got to do. That’s why I feel like some nurses just wouldn’t do it.” N4A, P. 291.* The transient work force impacted on requirements for ongoing education for rotated staff and agency nurses [27]. One study found it difficult to educate and train all shifts of nursing and assistants that may be involved as this changed daily [30], whist time and organisation for ongoing staff education were perceived as a significant barrier to implementation by others [27].

Some participants felt that the FWP would fit into their daily practices [27]: *“Just basic nursing care” N8C, P.291.* In contrast the fast pace, high turnover of patients and overall caseload impacted on nursing availability to follow through all recommendations for safe implementation, in particular oral care [20, 30]: *“That's going to be potentially another thing for nurses to have to do…they might be like, oh, well, it's just too hard, we're not going to give you your water or something. So if we're adding extra things for the nursing staff to do, I mean that could hinder the successfulness of the water protocol as well.” S DN3 P.639* [20]. Additional barriers were delegation of tasks to other staff for dependent patients, limited resources to record water intake [28, 29] and SLPs perceiving nurses prioritising other duties over implementing the FWP [28]: *“We expect nursing staff to do it, but they don’t always have time. Sometimes that gets put down lower on the priority list.” SLP2, P.115.*

The use of established systems to educate nurses about changes to current practices and processes to disseminate new protocols and staff expectations that they will learn new processes and procedures were factors that promoted implementation: *"We're always learning something new and we're always implementing new activities on the ward." N3A, P.290 *[27]*.* In one study SLPs provided education about the FWP guidelines to families and caregivers, and in house training to nursing staff and physicians before initiation of the FWP, for which attendance was documented [29]. Education involved skilled instructional education, demonstration and handout and teach back, and individualised education sessions to patients, caregivers, and nurses. Others reviewed the protocol with the patient and family, and nursing staff and nursing assistants [28]. Written material was provided, and nurses' competencies were checked after being educated. Peer support and modelling from supervisors and peers were identified as facilitators to implementation [20, 27]. *“So everything I did I talked about with my supervisor and she often prompted me…Having someone that was more experienced who could suggest when it would be appropriate, absolutely was helpful in confidence.” SLP6B, P.291* [27]*.* Not having a clear written protocol was the most significant barrier to implementation [20, 28].

Clinicians' attitude to risk aversion, their beliefs and previous experiences influenced clinical decision making and implementation of the FWP design [20]: *“As a general health service, we’re risk averse. And the logic of water and the FWP and the conditions you recommend water go against some of those built-in risk averse concerns that we have.” SSLP3, P.639.* The culture of routinely providing thickened fluids prevented clinicians considering the FWP. [20, 27].

### Domain IV: Individuals Characteristics Domain

Perceived facilitators were recipients’ quality of life through comfort, normalisation, preferences for care [20, 27] and hydration, although to a lesser degree [27]: *"I think it's important that we allow people to have the most normal life that they can have and if a little bit of water makes life more normal, then I think from that perspective it's quite an important thing.” DN4B, P.290* [27]. Not being ‘dry’ was a motivating factor for recipients; *"I gotta have the water…I'll dry up if I don't get the water" (*P3, P.117) [28]*.* Aspiration and development of chest complications were perceived negative outcomes, but not all participants were concerned with aspiration *"if the aspiration isn't developing into anything."* DN4B, P.290 [27]. Despite patients reporting their preference for water and SLPs stating patient choice was important, patient choice was often outweighed by factors associated with the patient’s medical condition and safety [20] with the focus only shifting to the person’s quality of life in palliative care or comfort situations.

Knowledge of the FWP intervention and a high degree of dysphagia expertise were perceived as implementation facilitators. Prior experience of the FWP made it quicker and easier to implement [27]. Greater experience was associated with increased confidence in the ability to determine patient suitability and implement the FWP in the acute setting [20]. Fewer years of experience were associated with more caution and risk avoidance. Knowledge and working with the stroke population was perceived to contribute positively to implementation [27]. In one study a stroke medical officer felt confident in trialing FWPs with all stroke patients if the patient was comfortable and not experiencing episodes of choking or excessive coughing. This was not a common opinion [20]. Clinicians’ understanding of stroke were perceived as factors that may perpetually reinforce the predominant practice patterns of not implementing the FWP in acute settings [20].

Lack of clarity of SLP instructions limited nursing staff ability to implement the FWP as intended. Incomplete and unclear documentation by nursing staff made it challenging for SLPs to monitor the impact of the FWP on the patient [28]. The sporadic and informal approach with which SLPs recommended the FWP was perceived to contribute to lack of awareness by other professionals [27]. Lack of stroke specific skills by new, student and agency nurses were concerns for misinterpretation of the FWP recommendations. The potential for misinterpretation also extended to family members. Most nurses were felt to lack the oral care skills required. Nurses forgetting to offer water and following measures to mitigate aspiration risk were examples where the FWP was not implemented as intended [28].

In one study almost two thirds of participants felt confident they could implement the FWP according to their role requirements and it was an extension of their current role [27]. Potential sources of implementation support were families to assist with positioning, oral care and monitoring outcomes. A common barrier was staff availability to deliver the intervention as intended. The level of staffing resource required to position and supervise patients, and the provision of oral care required, might preclude implementation [20, 27, 30]:*"But the bit that I don't see time for…is the mouth care and the rest of it. If they have to sit there and supervise sips of water, I can just see them [nursing] hand them a cup of water and walk out the door. …So steps are going to get missed."* DN2A, P.290 [27].

Deliverer and recipient receptivity and desire were motivating factors. Nurses discussed how they would be available for the FWP: *"I think we're obliged to make time. It's our job, and all of the allied health team have got their job to do, so personally I think you've got to make time"* SN5, P.640 [20]. Patients preferred and had a desire to have access to water [28]. Knowing that they could have water may help to counteract thirst and sense of dry mouth and encourage adherence to thickened fluids during mealtimes [20]. Positive feedback was reported from patients who received water [30].

Uncertainty about legal liability made nurses uncomfortable and less willing to implement the FWP [20, 27]. Negative staff attitudes about completing oral care [27] and patients refusing oral care were barriers to implementation. One study identified that SLPs may be reluctant due to the time it takes to set the patient up for the protocol with requirements such as: Getting doctor approval, obtaining patient consent, training patients, family and health care providers, putting up tracking sheets in patients’ rooms, and monitoring vitals daily [30]. Recipients' misgivings were about the discomfort and risk of pneumonia and water getting stuck. [28].

### Domain V: Implementation Process

The RCT study involved a phased implementation design comprising of: Phase 1 Candidacy, Phase 2 Education and Phase 3 Implementation [29]. The pilot feasibility study aimed to measure acceptability and fidelity, though the methods used for gathering recipient feedback about their health related QOL were unclear [30]. Several studies used interviews to identify perceived facilitators and barriers to FWP implementation [20, 27, 28]. In the mixed methods study information was also gathered from medical records [28]. Two studies [20, 28] explored decision making, one utilising the Situated Clinical Decision-Making Framework [32] to explore the complexity of clinical decision making and the decision-making process of clinicians about using the FWP. Another study [27] thematically analysed data and deductively mapped themes to the Theoretical Domains Framework [33]. The remaining studies did not use frameworks or models to assess context.

All health professionals were described as having a role in recommending, implementing and monitoring FWPs [20]. Leadership by senior clinicians and dedicated implementation leaders and champions were perceived as critical for change in practice and more widespread acceptance of the FWP in the acute stroke setting [20, 28]. Team support and availability of supervision often shaped decisions about patient suitability [20] and communication and collaboration among nurses, physicians and SLPs was felt to be necessary to monitor participant status [29]. Availability of family support in assisting with positioning and oral care and monitoring outcomes influenced some clinicians in their decision to implement a FWP [20]. The mindset of the FWP being the sole domain of SLPs was perceived to be a barrier to implementation: *"So whether they would see this as their responsibility—they might see it as a speechie [SLP] thing and think, not our [nursing] problem" [DN2A] P.289* [27]. Compared to other disciplines dieticians were considered to play a minimal role. Some SLP participants would not implement the FWP if they felt they could not “trust the family” P.638 [20].

Tailoring strategies included: implementation of oral care protocols before FWPs are implemented [29]; implementation materials including written materials, a tracking sheet for oral care and water intake to be kept at the patient’s bedside for staff and caregivers to fill out [30]; methods for communication and collaboration among nurses, physicians, SLPs to monitor patient status [29], individual education to implementation team members when necessitated by staff turnover [29]; providing the family with education to allow the FWP to be implemented more successfully (SLP1) [28] and daily monitoring of patients for signs and symptoms of aspiration including increased temperature, cough or congestion, positive chest X Rays and elevated white cell counts [30].

Two studies reflected on how an interdisciplinary approach and negotiation may facilitate implementation [27, 28]. One of these studies [27] combined the use of implementation champions and team members who work together to develop patient care plans. These plans detailed accountability for oral care, education, supervision, safe swallowing strategies, documentation, water provision and individual fluid requirements. Interdisciplinary negotiation involved clearly defined and documented roles and responsibilities of each discipline to allow for better integration of patient care across disciplines. Engagement with patients by *“Letting the patient know as well so that they’re aware that's something they can have …in case the nurses forget” SLP3. P116* [28] facilitated implementation.

Patients’ preference for water emphasised the importance of involving patients in the decision-making process [28]. The perceptions of patients and their families about how they weigh up choice versus safety has been recommended as the focus of future research [20]. Communicating preferences for care can be challenging for patients with communication and cognitive difficulties. All eligible patients should be involved in the decision-making process and information should be presented in an accessible format in their preferred language to enable them to make an informed choice. In patients who lack capacity, family and carers should be consulted and together with the stroke inter disciplinary team consider what would be in the patient’s best interests, particularly in those patients who dislike or who are refusing thickened fluids or when having thickened fluids or being NBM is negatively impacting on the person’s quality of life.

Observations of sub optimal oral care reinforced the need for development and implementation of oral care protocols in acute clinical settings before the FWP can be implemented successfully [27, 28]. Maintaining regular communication between clinicians, particularly at handover and continuing efforts to educate rotating and permanent members of staff on the FWP rules, risks and benefits would contribute to maximising adherence to patients recommended the FWP and minimise adverse patient outcomes [27]. Leadership and modelling of decision making and implementation by senior clinicians to influence attitude to risk and encourage use [20]. Offering water in lieu of thickened fluids between meals and training support staff and family members to offer and supervise patients who are recommended the FWP were ways of alleviating nurses’ workload [27].

A lack of implementation consensus and adaptations at multiple levels were identified [28]. Formalised guidelines and protocols for implementing the FWP detailing with whom, when and how to allow uptake were facilitators for implementation [20]. The uncertainty that the protocol would be implemented as intended may have contributed to SLP conservatism or risk aversion decision making [28]. The complexity of clinician decision making for patient suitability was identified [20]. Many reasons for exclusion were aligned to the exclusion criteria of research studies that have taken place in a different setting.

### Strength and Limitations of the Review

This systematic review is unique in its evaluation of the literature of the barriers and facilitators of the use of the FWP in the acute stroke setting. With a focus on the contextual determinants of implementation, the CFIR was selected as the implementation research framework to evaluate and map the data. This commonly used framework allowed for a systematic assessment of potential barriers and facilitators to implementation in our context. The search outcome retrieved a small number of studies, which was unsurprising given the focused review question and study selection criteria. However, with only five studies meeting the inclusion criteria, this limits the breadth and generalisability of the findings. The heterogeneity of study designs and small number of studies meant a quantitative meta-analysis was not possible and the review relied on qualitative narrative synthesis. There is also the potential risk of bias with multiple studies from the same research laboratory, which may introduce bias into the overall findings. One study [30] had several methodological limitations. However, in helping to answer the specific question addressed by this systematic review, the challenges identified in initiating and following through a FWP on an acute stroke care unit were both relevant and consistent with other included studies.

## Conclusion

Our systematic review has identified multiple interconnected contextual factors which may act as barriers or facilitators to FWP implementation in the acute stroke setting. Key barriers were a lack of evidence base and a standard protocol, trying to adapt and deliver a protocol designed for a different setting, complexity of patient selection and FWP design, culture of risk aversion, nursing staff availability and skills to deliver the FWP, and a greater use of agency nurses and transient workforce. Key facilitators to implementation were recommendations for research into its use, implementation of oral care protocols prior to implementing the FWP, the unique characteristics of the acute stroke unit setting, leadership and modelling by senior clinicians, interdisciplinary working and accountability for roles and responsibilities, regular communication and ongoing education and involving patients in the decision making and implementation. The findings from this review are the first stage of a feasibility study to investigate the acceptability, feasibility and fidelity of implementing the FWP in an NHS acute stroke unit setting. We will use lessons from this review and the CFIR to guide data collection for the next phase of the project which will inform the codesign of the implementation strategy and activities for FWP implementation.

## Supplementary Information

Below is the link to the electronic supplementary material.

Supplementary file1 (DOCX 132 KB)

## Data Availability

All data supporting the findings of this review are available within the paper and its Supplementary Information.
