# Supplementary material for: Barriers and Facilitators to implementation of the Free Water Protocol in the Acute Stroke Unit Setting: A Mixed Methods Systematic Review
Source: Dysphagia. 2025 Feb 5;40(5):1023–34. doi: 10.1007/s00455-025-10805-7 (PMC12479707; doi:10.1007/s00455-025-10805-7)
Supplement: Supplementary file 1 — Supplementary file1 (DOCX 132 KB) [file 455_2025_10805_MOESM1_ESM.docx]

**SUPPLEMENTARY MATERIAL**

**Title:** Barriers and Facilitators to implementation of the Free Water Protocol in acute stroke unit setting: A Mixed Methods Systematic Review

**Table 1:** Study Selection Criteria

**Table 2:** Example Search strategy - Medline

**Table 3:** Critical Appraisal Checklists

**Table 4:** Facilitators and barriers to implementation of the FWP in the acute stroke unit setting mapped on the CFIR

**Table 1: Study Selection Criteria**

| **Inclusion Criteria** | **Exclusion Criteria** |
| --- | --- |
| Stroke population | Non stroke population studies or mixed populations studies where information about stroke patients cannot be extrapolated from the published manuscript. |
| Free Water Protocol | Compensatory or other dysphagia rehabilitation approaches |
| Studies in Hospital Acute Stroke units. If the setting is not explicitly stated as ‘acute’ hospital stroke unit, outcome measures of studies will be reviewed for temporal indicators e.g., incidence of stroke-associated pneumonia or measures of hydration at 30 days compared to 3 months, which would be more indicative of a rehabilitation setting. | Non acute/step down rehabilitation units or long-term rehabilitation or domiciliary settings |
| Peer reviewed qualitative, quantitative mixed method studies. There are no restrictions on study design.  Studies in Systematic Reviews which meet inclusion criteria.  Grey literature. | Editorial Letters, book reviews, case reports. |

**Table 2: Example Search strategy - Medline**


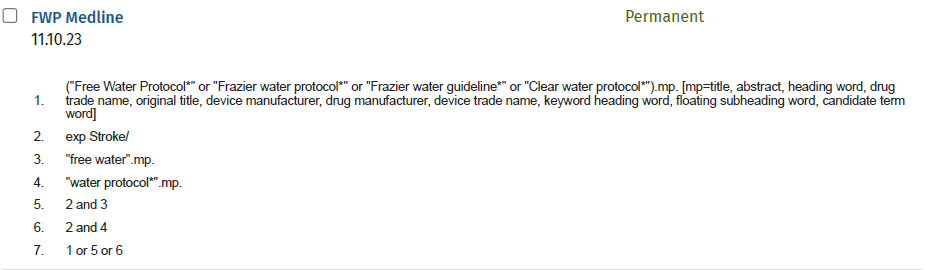


**Table 3: Critical Appraisal Checklists**

Barker A, Doeltgen S, Lynch E, Murray J. Perceived barriers and enablers for implementing water protocols in acute stroke care: A qualitative study using the Theoretical Domains Framework. Int J Speech Lang Pathol. 2019 Jun;21(3):286-294. doi: 10.1080/17549507.2019.1595145. PMID: 31213158.

| **Section A: Are the results valid?** | **Reviewer 1** | **Comments** | **Reviewer 2** | **Comments** |
| --- | --- | --- | --- | --- |
| 1. Was there a clear  statement of the aims of  the research? | Yes ✓ | To identify clinicians’ perceptions of enablers and barriers that may influence successful implementation of water protocols as an intervention for dysphagia in acute stroke unit settings. | Yes ✓ | Safety and efficacy of FWP in acute settings unclear as researchers have had difficulty recruiting participants and implementing water protocols in this setting – suggest barriers to implementation. Clear statement of aim ‘What are clinician’s perceptions of the enablers of and barriers to successful implementation of WPs in acute stroke care?’ |
|  | Can’t Tell |  | Can’t Tell |  |
|  | No |  | No |  |
| 2. Is qualitative  methodology  appropriate? | Yes ✓ | Qualitative research is the appropriate methodology to describe experiences from a subjective perspective of the research participants. | Yes ✓ | Qualitative descriptive approach to explore perceptions and experiences from the perspective of the individual. |
|  | Can’t Tell |  | Can’t Tell |  |
|  | No |  | No |  |
| 3. Was the research  design appropriate to  address the aims of the  research? | Yes ✓ | The researcher justified the research design and why it was appropriate for exploring the perceptions of the research participants. | Yes ✓ | Describe the qualitative approach as ideal for exploring perceptions and experiences from the perspective of the individual. Discuss theoretical domains framework as tool to identify enablers and barriers in healthcare settings. |
|  | Can’t Tell |  | Can’t Tell |  |
|  | No |  | No |  |
| 4. Was the recruitment  strategy appropriate to  the aims of the  research? | Yes ✓ | The researcher used a purposeful sample which included a range of disciplines involved in the implementation of the FWP and who had been employed for at least six months. Researchers explained how participants were identified across 3 tertiary hospitals by discipline managers who identified participants to ensure breadth of experience and perspectives. | Yes ✓ | Selection explained – recruited from 3 tertiary hospitals with acute stroke services in major Australian city. Purposive sampling from variety of disciplines. Needed to have been employed for at least 6 months and a range of junior-senior staff were included to ensure breadth of perspectives. No discussion as to whether anyone declined to take part. |
|  | Can’t Tell |  | Can’t Tell |  |
|  | No |  | No |  |
| 5. Was the data collected in  a way that addressed the  research issue? | Yes ✓ | The setting for the interviews was justified. Interviews took place at the participants' place of work, except 2 interviews conducted on the telephone due to time constraints. It is clear how the topic guide was developed. How data was collected (audio recorded) is clearly outlined, and the research discusses that 25-30 staff would meet data saturation. | Yes ✓ | The interview guide was collaboratively developed and mirrored Kallio et al. The guide was piloted twice to enable refinement. The semi structured interviews were audio recorded and the transcription and data checking were described. Two researchers simultaneously coded data and the research team regularly checked consensus on coding and generation of categories and themes. The authors talk about intention to meet data saturation but this was not achieved (discussed as a limitation). |
|  | Can’t Tell |  | Can’t Tell |  |
|  | No |  | No |  |
| 6. Has the relationship  between researcher and  participants been  adequately considered? | Yes | The researcher states that the interviews were conducted by 2 SLP students but does not reflect on their role or consider how they may have influenced sample recruitment or choice of location. Researcher Joanne Murray (co-author) conducted a lot of previous research on this topic which justifies the research question's formulation. | Yes | Interviews were conducted by 2 SLT students. There is no discussion as to any bias but rigor was increased by having 2 researchers simultaneously code the data. |
|  | Can’t Tell |  | Can’t Tell ✓ |  |
|  | No ✓ |  | No |  |
| **Section B: What are the results?** | **Reviewer 1** | **Comments** | **Reviewer 2** | **Comments** |
| 7. Have ethical issues been  taken into consideration? | Yes ✓ | Informed written consent was obtained and participants were given a pseudonym to maintain confidentiality. Approval has been sought from the relevant REC. | Yes ✓ | Ethical approval provided. Informed written consent was obtained and pseudonyms were used to maintain confidentiality. |
|  | Can’t Tell |  | Can’t Tell |  |
|  | No |  | No |  |
| 8. Was the data analysis  sufficiently rigorous? | Yes ✓ | There is an in-depth description of analysis. Data was inductively thematically analysed using Braun & Clarke approach and coded. Themes were then deductively mapped onto the TDF framework. The researcher explains how the data was selected. Two researchers checked the interview transcripts and transcripts were returned to participants for member checking. Researchers explain how the research team checked consensus on coding and generation of categories and themes. | Yes ✓ | Data analysis is clearly described (see pt 288). Quotes used throughout (and supplementary material available). Contradictory data not discussed. No discussion of potential bias of researchers  . |
|  | Can’t Tell |  | Can’t Tell |  |
|  | No |  | No |  |
| 9. Is there a clear statement  of findings? | Yes ✓ | The findings are clearly presented and there is adequate discussion about reported enablers and barriers to implementation of the FWP. The researcher has discussed steps to ensure rigour and the findings are discussed in relation to the research question. | Yes ✓ | Findings are clearly presented and illustrated with examples - clear to state whether thoughts were evident for some/ all of the participants and linked this to participant role. Don't discuss triangulation. The findings are discussed in relation to original questions |
|  | Can’t Tell |  | Can’t Tell |  |
|  | No |  | No |  |
| **Section C: Will the results help locally?** | **Reviewer 1** | **Comments** | **Reviewer 2** | **Comments** |
| 10. How valuable is the  research? | N/A | The researcher discussed the contribution the study makes to existing knowledge and in relation to current practice (communication and education; oral care; nursing workload; aversion to aspiration and interdisciplinary implementation). They identify that future research is needed to test the safety and efficacy of WPs in acute stroke. They have considered how the findings may translate to other dysphagia management practices and implementation of guidelines and protocols in other complex practice areas. | N/A | The authors discuss the range of barriers and enablers to influence the implementation of the FWP in acute stroke care in relation to current practice. They discuss the need to address these barriers and optimise enablers to opimise uptake of the FWP in this setting. They consider findings in relation to implementing guideline/ protocols in other complex practice areas. |

Murray J, Maloney S, Underdown K, Doeltgen S. Patient suitability for free water protocols in acute stroke and general medicine: a qualitative study of clinician perceptions. Int J Lang Commun Disord. 2022 May;57(3):630-644. doi: 10.1111/1460-6984.12713. Epub 2022 Mar 23. PMID: 35318783.

| **Section A: Are the results valid?** | **Reviewer 1** | **Comments** | **Reviewer 2** | **Comments** |
| --- | --- | --- | --- | --- |
| 1. Was there a clear  statement of the aims of  the research? | Yes ✓ | The research aims to explore which patients with dysphagia clinicians consider suitable, or not for the FWP, and why. | Yes ✓ | The goal was to explore the perceptions and decision-making process of clinicians about using FWP. Important as there is a gap in research on FWP in acute care settings ? is this due to higher complexity of patients meaning they did not meet inclusion criteria OR nature of acute setting rendering FWP implementation unfeasible. |
|  | Can’t Tell |  | Can’t Tell |  |
|  | No |  | No |  |
| 2. Is qualitative  methodology  appropriate? | Yes ✓ | Qualitative research is the appropriate methodology to describe experiences from a subjective perspective of the research participants. | Yes ✓ | Qualitative methodology appropriate as wish to conduct in depth exploration of perspectives. |
|  | Can’t Tell |  | Can’t Tell |  |
|  | No |  | No |  |
| 3. Was the research  design appropriate to  address the aims of the  research? | Yes ✓ | The researcher has not justified the research design, although it is appropriate for exploring the research participants' perspectives. | Yes ✓ | No justification of research design but clear that the goal was to explore views and perceptions of clinicians in the acute care setting and this was the appropriate methodology to use for this purpose. |
|  | Can’t Tell |  | Can’t Tell ✓ |  |
|  | No |  | No |  |
| 4. Was the recruitment  strategy appropriate to  the aims of the  research? | Yes ✓ | The researcher used a purposeful sampling technique to identify and recruit participants from the multidisciplinary management team who are most likely involved in the decision making, oversight and implementation of the FWP in the research settings. They explain how decision makers were involved with the identification of possible participants to ensure a breadth of perspectives and experiences were recruited. | Yes ✓ | Clear description of how participants were selected (purposive sampling from 3 tertiary hospitals). The authors describe why the disciplines were selected as they represent members of the MDT who would most likely be involved in decision making, oversight and implementation of FWP. Ensured breadth of experience.  There was no discussion as to whether anyone decided not to take part. |
|  | Can’t Tell |  | Can’t Tell |  |
|  | No |  | No |  |
| 5. Was the data collected in  a way that addressed the  research issue? | Yes ✓ | The settings for the interviews were convenient for the participants. It is clear how the data was collected i.e. via audio recorded, individual semi structured interviews face to face or via the telephone. The researcher has not justified the methods chosen but has explained how they developed the topic guide. The form of data is clear - 20 to 50 min audio recordings. The researcher does not discuss saturation of data but acknowledges that the study is limited by the small number of participants. | Yes ✓ | Semi structured interviews were conducted either face: face or on phone. The interviews were audio recorded.  Transcripts were checked against audio recording and with participants.  The interview guide was collaboratively developed and followed a staged approach that closely mirrored Kallio et al. pilot interviews conducted to test and refine questions. Note there was no discussion about the saturation of data. |
|  | Can’t Tell |  | Can’t Tell |  |
|  | No |  | No |  |
| 6. Has the relationship  between researcher and  participants been  adequately considered? | Yes | The researcher does not critically examine their own role, potential bias, and influence during the formulation of the research questions*, data collection (including sample recruitment and choice of location). * The lead author Joanne Murray has conducted previous research on this topic which justifies the formulation of the research question. | Yes | There is no description of the 'research team' who conducted the interview or their profession/ background/ potential bias. |
|  | Can’t Tell |  | Can’t Tell |  |
|  | No ✓ |  | No ✓ |  |
| **Section B: What are the results?** | **Reviewer 1** | **Comments** | **Reviewer 2** | **Comments** |
| 7. Have ethical issues been  taken into consideration? | Yes ✓ | Informed written consent was obtained and participants were given a pseudonym to maintain confidentiality. Approval has been sought from the relevant REC. | Yes ✓ | Ethical approval obtained and detailed. Details are provided about how the research was explained to participants (standard info provided re FWP), and written consent was obtained. |
|  | Can’t Tell |  | Can’t Tell |  |
|  | No |  | No |  |
| 8. Was the data analysis  sufficiently rigorous? | Yes ✓ | There is an in-depth description of the analysis. Data was analysed using the first four stages of the Braun & Clarke (2013) thematic analysis process. The researcher derived the categories from the data by inductively line by line coding and searching for categories by matching like with like codes and establishing relationships between them. These categories were then mapped deductively into the existing themes of the Situated Clinical Decision-Making Framework (Gillespie & Petersen, 2009). There is sufficient data to support the findings, including supplementary material. The researchers discuss differences in clinician’s perceptions. The researcher does not acknowledge potential bias or influence of their role. | Yes ✓ | Interview data were analysed using thematic analysis (Braun and Clarke, 2013). Categories were mapped onto the CDF framework. Researchers identified and reached a consensus. The authors don’t state how many researchers conducted the analysis. Quotes are provided to illustrate. There is no examination of researcher's role or potential bias and influence during analysis |
|  | Can’t Tell |  | Can’t Tell |  |
|  | No |  | No |  |
| 9. Is there a clear statement  of findings? | Yes ✓ | The findings are clearly presented under the different dimensions of the CDF and are discussed in relation to the original research question. The research does not discuss the credibility of their findings from the perspective of triangulation or more than one analyst but does acknowledge the limitations of the size of the study, self-reported practices from a hypothetical or historical perspective, social desirability bias and lack of patient perspective. The researchers do not discuss evidence against the researchers’ arguments other than described in the limitations section. | Yes ✓ | A thorough description of findings is presented and discussed in relation to the research question.  Researchers not making an argument per se, just presenting the findings with respect to the framework used - they reported in some instances whether the reports were common or 'not a common opinion of participants'.  There is no discussion re credibility of findings or triangulation. |
|  | Can’t Tell |  | Can’t Tell |  |
|  | No |  | No |  |
| **Section C: Will the results help locally?** | **Reviewer 1** | **Comments** | **Reviewer 2** | **Comments** |
| 10. How valuable is the  research? | N/A | The researcher discusses what the research adds to existing knowledge: SLPs are the gate keepers; decision making process is complex; disease conditions and illnesses often used as exclusionary criteria; decision making privileged risk aversions over patient preference in most settings, except for palliative care. Lack of clinical guidelines and research evidence in acute settings and focus on risk aversion perpetuate avoidance of FWP in acute settings and leadership by senior clinicians appears critical for change in practice in this space. | N/A | The authors consider how findings may explain the lack of implementation of FWP in acute settings. They consider areas where further research is necessary - i.e. need for multi-site trials with broader consideration of patient function rather than using diagnostic based exclusion criteria along with identification of modifiable context specific barriers. They discuss the need for the development of clear FWP implementation guidelines for use in the acute care setting. They discuss the lack of evidence for safety of FWP in acute care settings. |

Kenedi H, Campbell-Vance J, Reynolds J, Foreman M, Dollaghan C, Graybeal D, Warren AM, Bennett M. Implementation and Analysis of a Free Water Protocol in Acute Trauma and Stroke Patients. Crit Care Nurse. 2019 Jun;39(3):e9-e17. doi: 10.4037/ccn2019238. PMID: 31154338.

| **Section A: Is the basic study design valid for a randomised controlled trial?** | **Reviewer 1** | **Comments** | **Reviewer 2** | **Comments** |
| --- | --- | --- | --- | --- |
| 1. Did the study address a clearly focused research question? | Yes ✓ | The study was designed to assess the outcomes of an intervention. The research question was focused in terms of: Population studied - English or Spanish adults aged 18 >; admittance to an acute care unit; documented aspiration on thin liquids according to an objective examination and mental status able to support oral intake of thin liquids; Intervention, Comparator - control group with no access to thin fluids and Outcomes; 3 positive clinical outcomes (number of diet upgrades, fewer days to diet upgrade, and fewer days in the study) and 3 negative clinical indicators (intubation, pneumonia, and diet downgrade) in the acute care setting and monitoring participants discharged into the hospital system's rehabilitation setting. | Yes ✓ | Population studied patients with dysphagia who aspirated thin fluids, aetiology either stroke or trauma. Intervention group followed the FWP. Comparator – control group with no access to water. Outcomes measured – 3 positive clinical indicators (no. of diet upgrades, fewer days to upgrade, fewer days in study) and 3 negative clinical indicators (intubation, pneumonia, diet downgrade). Study conducted in acute care setting. |
|  | Can’t Tell |  | Can’t Tell |  |
|  | No |  | No |  |
| 2. Was the assignment of participants to interventions randomised? | Yes ✓ | Participants were randomised to the control group or experimental group via odd and even numbers from a sealed envelope. Randomisation was felt sufficient to eliminate bias. Study personnel who entered, audited and analysed the data were blinded to group assignment. Not possible to conceal/blind participants or onsite researchers. | Yes ✓ | Participants were randomised to groups by odd and even numbers from a sealed envelope. The randomisation was sufficient to eliminate bias. It was not possible to blind participants or onsite researchers to the group allocation. |
|  | Can’t Tell |  | Can’t Tell |  |
|  | No |  | No |  |
| 3. Were all participants who entered the study accounted for at its conclusion? | Yes ✓ | Losses to the study were accounted for: 2 participants from the experimental group did not complete the study; 1 participant was discharged before data collection and 1 participant died for reasons unrelated to the study. Participants were analysed in the study groups to which they were randomised. The study did not stop early. Unable to separate stroke patients vs. trauma patients in terms of outcomes measured. | Yes ✓ | Losses to follow up and exclusions after randomization were accounted for. Participants were analysed in the study groups to which they were randomised. The study was not stopped early. |
|  | Can’t Tell |  | Can’t Tell |  |
|  | No |  | No |  |
| **Section B: Was the study methodologically sound?** | **Reviewer 1** | **Comments** | **Reviewer 2** | **Comments** |
| 4. Were the participants ‘blind’ to intervention they were given? Were the investigators ‘blind’ to the intervention they were giving to participants? Were the people assessing/analysing outcome/s ‘blinded’? | No ✓ | It was not possible to blind participants to the intervention they were given. | No ✓ |  |
| 5. Were the study groups similar at the start of the randomised controlled trial? | Yes | There were no significant differences between control and experimental group for age, sex, diagnosis, pneumonia or dehydration. There was a significant difference in patients with a tracheostomy speaking valve in the experimental group. | Yes ✓ | Baseline characteristics of each study group were clearly set out and there were no significant differences between the groups in terms o age, sex, diagnosis, pneumonia or dehydration. There were more pts with a tracheostomy speaking valve in the experimental group. |
|  | Can’t Tell |  | Can’t Tell |  |
|  | No ✓ |  | No |  |
| 6. Apart from the experimental intervention, did each study group receive the same level of care (that is, were they treated equally)? | Yes ✓ | Eligible patients in both groups received modified diets (by mouth or enteral feeding) and dysphagia management consistent with national best practice guidelines and the hospital's standard clinical protocol which included facilitative oropharyngeal exercises, compensatory strategies and dietary modification including thickened liquids during meals and oral medication and the Aspiration Precaution Oral Care Program. In the experimental group, the FWP guidelines and implementation design of the study were clearly outlined. Yes, the follow up intervals for the subset of participants were followed up until discharge from rehabilitation were the same for both groups. | Yes ✓ |  |
|  | Can’t Tell |  | Can’t Tell |  |
|  | No |  | No |  |
| **Section C: What are the results?** | **Reviewer 1** | **Comments** | **Reviewer 2** | **Comments** |
| 7. Were the effects of intervention reported comprehensively? | Yes ✓ | A power calculation was undertaken to determine 53 patients per group and a significance level of 0.05. 3 positive clinical outcomes (number of diet upgrades, fewer days to diet upgrade, and fewer days in the study) and 3 negative clinical indicators (intubation, pneumonia, and diet downgrade) in the acute care setting and monitoring participants discharged into the hospital system's rehabilitation setting. P values were reported. Results were reported for each outcome for the experimental and control group in the acute setting. Logression analysis and multiple regression analysis was performed to analyse the positive clinical outcomes. Patient demographics, clinical information, positive clinical outcomes and negative clinical indicators were compared using t tests or Wilcoxon tests for numerical variables, log-rank tests for time to upgrade, and X2 or Fisher tests for categorical variables. There was no missing or incomplete data or differential drop out between the study groups that could affect the results. Potential limitations were identified - low statistical power, duration of follow up re development of pneumonia. | Yes ✓ | A power calculation was undertaken which revealed that 53 pts were required per group (see pg 14). Outcomes were measured, and were they clearly specified- No of diet upgrades, fewer days to diet upgrade, fewer days in study, intubation, pneumonia and diet downgrade. Results were reported for each outcome in each study group at each follow-up interval. There was no missing or incomplete data. There was no differential drop-out between the study groups that could affect the results. Potential sources of bias were not identified. Statistical tests were used but not described. P values were reported. |
|  | Can’t Tell |  | Can’t Tell |  |
|  | No |  | No |  |
| 8. Was the precision of the estimate of the intervention or treatment effect reported? | Yes ✓ | P values were reported and confidence intervals were reported. | Yes ✓ | . |
|  | Can’t Tell |  | Can’t Tell |  |
|  | No |  | No |  |
| 9. Do the benefits of the experimental intervention outweigh the harms and costs? | Yes | There were no significant group differences in the 3 positive indicators. 1 patient in the experimental group experienced a diet downgrade. A cost effectiveness analysis was not undertaken. Adverse events were reported but not all were reported for each study group. | Yes | There were no significant group differences in the 3 positive indicators. Harms or unintended effects were reported for each study group. A cost effective analysis was not undertaken. |
|  | Can’t Tell ✓ |  | Can’t Tell ✓ |  |
|  | No |  | No |  |
| **Section D: Will the results help locally?** | **Reviewer 1** | **Comments** | **Reviewer 2** | **Comments** |
| 10. Can the results be applied to your local population/in your context? | Yes ✓ | The non tracheostomy stroke participants are like the participants in the local population. Secondary diagnoses of pneumonia and dehydration are important to our study population. Other outcomes of interest would be patient satisfaction of the FWP and implementation outcomes of introducing the FWP. Some information was presented separately for stroke patients but not the positive and negative clinical indicators. | Yes ✓ | The study participants include those who are similar to the patients in our care, but they also included patients with dysphagia secondary to trauma. It would have been useful if findings had been split into stroke/ non stroke populations. Limitations of the study include short duration of stay in acute setting. The main limitation for our systematic review is pulling out data specific to stroke. |
|  | Can’t Tell |  | Can’t Tell |  |
|  | No |  | No |  |
| 11. Would the experimental intervention provide greater value to the people in your care than any of the existing interventions? | Yes | The FWP could enhance patient care in the acute setting, but there is a need to further establish the evidence base for implementing the FWP in the acute setting. Potential challenges of implementing the FWP in the acute setting have been identified - short stays - participants were monitored for an average of 6 days after enrollment. Future research will require adherence to oral care and FWPs and education of MDT members. | Yes ✓ |  |
|  | Can’t Tell ✓ |  | Can’t Tell |  |
|  | No |  | No |  |

Weber V. The challenges of initiating the Frazier Water Protocol on an acute care stroke unit. Institute for Nursing Newsletter. 2009;5(3):10.

| **Section A: Are the results valid?** | **Reviewer 1** | **Comments** | **Reviewer 2** | **Comments** |
| --- | --- | --- | --- | --- |
| 1. Did the study address a clearly  focused issue? | Yes ✓ | The research had 3 focused questions: Does implementation of the FWP result in balance between intake and output in acute stroke patients? How well do patients adhere the FWP? How will patients who participate in the FWP view their health QOL? | Yes ✓ | Setting is specified as acute stroke unit. Does the implementation of the FWP result in balance between intake and output? How well do patients adhere to the FWP? How will pts in the FWP view their health related QOL? |
|  | Can’t Tell |  | Can’t Tell |  |
|  | No |  | No |  |
| 2. Was the cohort recruited in  an acceptable way? | Yes ✓ | All patients are evaluated by the SLP to determine if they meet the inclusion criteria. Exclusion criteria are defined. 5 out of 20 patients recruited at time of publication. Everybody is included who should have been. | Yes ✓ | All patients on unit were evaluated by SLT to see if they met inclusion/ exclusion criteria. Goal was 20 but at time of publication only 5 pts had been recruited. |
|  | Can’t Tell |  | Can’t Tell |  |
|  | No |  | No |  |
| 3. Was the exposure accurately  measured to minimise bias? | Yes ✓ | FWP guidelines were reviewed with the patient, family, nursing staff and assistants for each patient. A tracking sheet for oral care and water intake was kept at the patient's bedside to fill out for fluids intake. All subjects were receiving the same FWP. | Yes ✓ | All eligible pts were placed in exposure (FWP) group in the same way. A tracking sheet was kept at pt’s bedside to monitor fluid intake and to monitor pt for adverse effects (increased temp, increased cough, chest xray or elevated white cell count). |
|  | Can’t Tell |  | Can’t Tell |  |
|  | No |  | No |  |
| 4. Was the outcome accurately  measured to minimise bias? | Yes | A tracking sheet for oral care and water intake was kept at the patient's bedside to fill out for fluids intake. Can't tell how output is measured. Can't tell what measurements were used to measure patient adherence or how QOL was measured. Can't tell if the outcome assessor was blinded to the FWP. | Yes | The outcome measures were discussed but no results presented in the paper. The paper discusses the challenges presented during the study in recruiting and completing the protocol. |
|  | Can’t Tell ✓ |  | Can’t Tell |  |
|  | No |  | No ✓ |  |
| 5. (a) Have the authors identified  all important confounding  factors? | Yes | Potential for confounding factors such as accuracy/reliability of measuring balance between fluid intake and output in different settings e.g., home, not accounted for. Did not account for patients being transferred out of district and availability of follow up. Did not provide participant characteristics therefore unable to identify potential confounding factors. | Yes | Outcomes should have been amount of water taken and signs/ symptoms of aspiration pneumonia. No patient characteristics discussed so unable to identify confounding factors - i.e. level of dependency for drinking water, chest vulnerability, mobility ability to self feed, condition of mouth etc. |
|  | Can’t Tell |  | Can’t Tell ✓ |  |
|  | No ✓ |  | No |  |
| 5. (b) Have they taken account of  the confounding factors in the  design and/or analysis? | Yes | Minimal analysis/results have been presented. Authors have identified challenges of initiating FWP. They have considered nurse training in the design stage but did not consider potential for staff changes and how adherence would be monitored. Only 5 patients out of target 20 recruited over 7 months potential for confounding related to recruitment bias i.e. patients who meet inclusion criteria but who are not considered suitable to go forward to be on the FWP. | Yes | No results presented in study - discusses reasons for this in relation to difficulty recruiting to the study. |
|  | Can’t Tell |  | Can’t Tell |  |
|  | No ✓ |  | No ✓ |  |
| 6. (a) Was the follow up of  subjects complete enough? | Yes | The study is in progress. Participants have been lost to the study who are transferred out of the network. Potential for those participants to have different outcomes. | Yes |  |
|  | Can’t Tell |  | Can’t Tell |  |
|  | No ✓ |  | No ✓ |  |
| 6. (b) Was the follow up of  subjects long enough? | Yes | The study is in progress. Potential for a large proportion of the patients to be upgraded to regular fluids earlier than 3 months/prior to discharge. | Yes | No discussion of follow up of patients as no findings presented |
|  | Can’t Tell |  | Can’t Tell |  |
|  | No ✓ |  | No ✓ |  |
| **Section B: What are the results?** | **Reviewer 1** | **Comments** | **Reviewer 2** | **Comments** |
| 7. What are the results of this study? |  | The study is in progress and limited outcome measurement data has been presented. All 5 participants recruited so far felt their medical QOL improved and there has been no medical compromise so far. Challenges that have occurred so far have been presented. |  | It is difficult to recruit to FWP in the acute care setting. The reasons for this are discussed: pts are often discharged quickly, difficulties training all staff, staff ++ busy, SLT may perceive it involves too much set up. Pt may improve and be upgraded to L0 fluids. |
| 8. How precise are the results? |  | The study is in progress. No CI’s are presented. |  | N/A |
| 9. Do you believe the results? | Yes ✓ | Due to the study still being in progress can't tell yet but the challenges described are consistent with the available evidence. | Yes ✓ | The author is unable to address the research questions posed in this study due to difficulties recruiting to the study. They discuss reasons for this which in themselves are valuable. |
|  | Can’t Tell |  | Can’t Tell |  |
|  | No |  | No |  |
| **Section C: Will the results help locally?** | **Reviewer 1** | **Comments** | **Reviewer 2** | **Comments** |
| 10. Can the results be applied to  the local population? | Yes ✓ | The study design is appropriate. The study participants and setting are the same as the local population. The challenges faced in the acute setting can applied to the local population. | Yes ✓ | It is likely that all acute stroke units would face similar challenges. |
|  | Can’t Tell |  | Can’t Tell |  |
|  | No |  | No |  |
| 11. Do the results of this study fit  with other available  evidence? | Yes ✓ | The findings so far in terms of the limited results available are consistent with other studies that the FWP improves QOL without having a detrimental effect on medical compromise. The challenges of initiating a FWP in an acute stroke unit setting are consistent with other studies. | Yes ✓ | In terms of discussions of barriers to implementing the FWP in the acute care setting the findings are consistent with other studies who also failed to recruit in this setting. |
|  | Can’t Tell |  | Can’t Tell |  |
|  | No |  | No |  |
| 12. What are the implications of  this study for practice? | Yes | The study is in progress but the challenges identified so far are consistent with implementation challenges that have been identified in the acute setting and are relevant to the systematic review question. | Yes | This study was unable to address the research questions it set out to but provides valuable insight into the barriers of implementing the FWP and conducting research re the FWP in the acute stroke setting. It is felt that these identified factors are a useful contributor to research in this field and that the paper should be included in the systematic review - albeit being aware that it is the implementation of the FWP that is discussed rather than outcome measures pertaining to the safety or potential benefits of the FWP in this setting. |
|  | Can’t Tell ✓ |  | Can’t Tell ✓ |  |
|  | No |  | No |  |

Murray J, Walker C, Doeltgen S. Implementation of free water protocols in acute care: An observation of practice. Int J Speech Lang Pathol. 2022 Apr;24(2):111-121. doi: 10.1080/17549507.2021.1955973. Epub 2021 Aug 3. PMID: 34343448.

| **Screening questions** | **Reviewer 1** | **Comments** | **Reviewer 2** | **Comments** |
| --- | --- | --- | --- | --- |
| 1. Are there clear research questions? | Yes ✓ | 1. How do SLPs arrive at a recommendation relating to the FWP i.e. decision making about patient suitability? 2. How do nursing staff implement the FWP? 3. What are the perceived barriers and enablers by SLPs and nursing to implementing the FWP? 4. What are patient's experiences of the FWP (preferences or concerns)? | Yes ✓ | Research questions clear: To observe how and with whom FWP are implemented in acute stroke and general medical units. |
|  | Can’t Tell |  | Can’t Tell |  |
|  | No |  | No |  |
| 2. Do the collected data allow to address the research questions? | Yes ✓ | The study contributes new insights, but some findings were limited by incomplete documentation not accurately representing patient outcomes, and small interview triads. | Yes ✓ | Collected data address the research questions – for a small sample. |
|  | Can’t Tell |  | Can’t Tell |  |
|  | No |  | No |  |
| **Methodological Quality Criteria** | **Reviewer 1** | **Comments** | **Reviewer 2** | **Comments** |
| 5 i. Is there an adequate rationale for using a mixed methods design to address the research question? | Yes ✓ | A mixed methods design was employed to enable exploration of FWP implementation from different perspectives. Comparison of information from documented (medical records, nursing plan, and purpose made data collection tool), reported (interviews) and observed care (HPC, functional status, dysphagia status, vital signs, fluid intake) to facilitate understanding of current practice. | Yes ✓ | Collected data from semi-structured interviews (qualitative) and a review of medical records and nursing plans (Quantitative). Information gathered around the decision making process, how tasks were delegated, what tasks were completed, how frequently and by whom, pt presentation including demographics, preliminary outcomes -amount of water offered and consumed, observations, blood tests and urine analysis. From this gave a detailed description of who the FWP was used with, how it was implemented, and the challenges it posed. To raise awareness of current utilisation of FWP in acute care and encourage readers to reflect on their own decision making. |
|  | Can’t Tell |  | Can’t Tell |  |
|  | No |  | No |  |
| 5 ii. Are the different components of the study effectively integrated to answer the research question? | Yes ✓ | The results from each of the methods were integrated during the interpretation phase. Information was gathered from both methods in a summary, and the data was jointly displayed. | Yes ✓ | A summary of information obtained from medical records and from interviews is presented, and shows some inconsistencies, which are discussed in the text. Methods were ‘prioritised equally’ and data collection was concurrent with methods kept independent during collection and analysis and integrated during interpretation phase. |
|  | Can’t Tell |  | Can’t Tell |  |
|  | No |  | No |  |
| 5 iii. Are the outputs of the integration of qualitative and quantitative components adequately interpreted? | Yes ✓ | Meta inferences occurred in the interpretation of the data which were discussed and visually represented in a figure showing the interaction of factors affecting implementation. | Yes ✓ | The outputs are discussed for each individual case study. Interesting that the individuals did not meet criteria for FWP (i.e. not necessarily aspirating water) and that the protocol was not followed as per standard (i.e. restricting to teaspoons) but this was perceived to be following the FWP which in itself is interesting. |
|  | Can’t Tell |  | Can’t Tell |  |
|  | No |  | No |  |
| 5 iv. Are divergences and inconsistencies between quantitative and qualitative results adequately addressed? | Yes ✓ | There was visual side-by-side display of quantitative and qualitative data including differences between the two data sources. Examples of convergence and divergence were also discussed in the Discussion section and explanations proposed. | Yes | There is discussion about how the patients and protocols differed from the standard FWP. The inconsistencies between information collected via quanitiative and qualitative methods are discussed. |
|  | Can’t Tell |  | Can’t Tell ✓ |  |
|  | No |  | No |  |
| 5 v. Do the different components of the study adhere to the quality criteria of each tradition of the methods involved? | Yes ✓ | Use of existing records as a data source meant there were limitations in the accuracy of available documentation and reporting. A purposeful data collection tool was created to record observations of practice. Interview transcripts were analysed thematically using Braun & Clarke thematic process. Quotations were provided to justify the themes. There were no reflections by the research team of potential bias or techniques to enhance trustworthiness. | Yes ✓ | Yes, although some details were missing.  Purposive sampling, clear inclusion/ exclusion criteria.  The data collection tool is described. Semi structured interviews were recorded, and the guide had been developed and revised - available in supplementary material. The interview transcripts were analysed thematically – doesn’t talk about whether data saturation was reached or if independently analysed by researchers – codes ‘checked’ by research team. The quantitative data were reported in the text but as there were no standards for when the data were collected (i.e. it was a case note analysis) this cannot be compared between pts – just trends within and btw patients. |
|  | Can’t Tell |  | Can’t Tell |  |
|  | No |  | No |  |

**Table 4: Facilitators and barriers to implementation of the FWP in the acute stroke unit setting mapped on the CFIR**

1. **INNOVATION DOMAIN**

| **Construct Name and Construct Definition** | **Facilitator** | **Barrier** |
| --- | --- | --- |
| 1. **Innovation Source – The degree to which the group that developed and/or visibly sponsored use of the innovation is reputable, credible, and/or trustable.**   Updated CFIR: Interview Guide Questions & Coding Guidelines (June 2023) - **Include statements about:** · The type of innovation source, e.g., external sources, including academic, governmental, or commercial entities; internal sources, including individuals or groups in the Inner Setting; and/or external/internal sources, where the innovation was co-developed and/or sponsored. · Characteristics of the innovation developers **Exclude statements about:** • Implementation support and/or facilitation that may be provided by the Innovation Source, and instead code to Individuals: Implementation Facilitators or another appropriate Role. | - **Reputation and credibility of the FWP** - All included studies were consistent in referencing ‘The Frazier Free Water Protocol’ developed by Kathy Panther at the Frazier Rehabilitation Insitute, Kentucky, USA, as the innovation source. Terminology used by studies: Water Protocols (WPs) (Barker 2019), Free Water Protocol (Kenedi 2019, Murray 2022a, 2022b), Frazier Water Protocol (Weber 2009). |  |
| 1. **Evidence Base – The degree to which the innovation has robust evidence supporting its effectiveness**   Updated CFIR: Interview Guide Questions & Coding Guidelines (June 2023) - **Include statements about**: · Different types and sources of evidence, e.g., published literature, guidelines, anecdotal stories from colleagues, information from a competitor, previous experiences with recipients or from a pilot. · Any evidence demonstrating that the innovation will (or will not) address the underlying problem. **Exclude statements about**: • Actual innovation outcomes in the Inner Setting e.g., in a retrospective evaluation, a statement such as "The innovation was effective for our patients," and instead code to Innovation Outcomes: Recipient Impact (see CFIR Outcomes Addendum). • Sharing evidence to engage individuals in implementation and/or delivery of the innovation, and instead code to Implementation Process: Engaging. |  | - **Lack of evidence base in the acute stroke setting** - Although positive findings support the use of the WPs in the rehabilitation stage following stroke, safety and efficacy in acute setting remains unclear **(**P.287) – Incorporating WPs into hospital policies and procedures “*is unlikely to occur until research has shown WPs to be safe and effective for this patient population.*” (p.292) Barker et al. (2019); Knowing that the evidence for the safety of the FWP in acute settings has been (under) explored may have impacted on SLP selection of patients with low risk of aspirating water because of their high medical acuity and probably poor host resistance (p. 118) Murray et al. (2022b); Lack of empirical evidence (e10) concerning the FWP particularly in the acute setting provided the rationale for the study **>** *Larger scale studies are needed to reach decisive conclusion on the positive and negative indicators of a FWP in the acute setting.(e17)* Kenedi et al (2019). - **Lack of clinical guidelines -** *Lack of clinical guidelines and research evidence* in acute settings (and a focus on risk aversion) seem to reinforce the avoidance of the FWP in acute settings (p.631) Murray et al. (2022a). |
| 1. **Relative advantage – The degree to which the innovation is better than other available innovations or current practice.**   Updated CFIR: Interview Guide Questions & Coding Guidelines (June 2023) - **Include statements about**: • Various types of advantages/disadvantages, e.g., at the system-level (e.g., financially dis(advantageous) to the Inner Setting) or individual-level (e.g., clinically dis(advantageous) for Deliverers/Recipients). **Exclude statements about**: • The extent to which the innovation is needed, and instead code to Tension for Change. | - **Clinically advantageous for recipients –** Based on 5 patients who had been initiated into the study at the time of publication - Positive feedback with patients feeling their medical QOL improved. None of the patients had any medical compromise Weber (2009) - **‘Non inferior outcomes’** - Kenedi et al. in Murray et al. (2022b, p.111). Acknowledging limitations for statistical power, Kenedi et al (2019) reported no significant group differences for eligible stroke and trauma patients in positive outcomes, and negative clinical indicators did not differ significantly between the control and experimental groups. Compared to trauma patients, stroke patients took longer and were less likely to have a diet upgrade. |  |
| 1. **Adaptability - The degree to which the innovation can be modified, tailored, or refined to fit local context or needs**   Updated CFIR: Interview Guide Questions & Coding Guidelines (June 2023) - This construct captures the inherent adaptability of the innovation, not the need to adapt nor the process of adapting the innovation. I**nclude statements about:** • The (in)ability to adapt the innovation, e.g., complaints about the rigidity of the protocol, due to features of the innovation itself or lack of “permission” to change components. **Exclude statements about:** • Adapting the innovation, and instead code to Implementation Process: Adapting. • The innovation needing/not needing to be adapted, and instead code to the relevant CFIR construct, e.g., if an innovation needs/needed to be adapted because it did not meet recipients’ needs, code to Innovation Recipient: Needs; if an innovation needs/needed to be adapted because it did not fit with existing workflows, code to Compatibility. | - Weber (2009) *“The Frazier Water Protocol has been* ***tailored*** *to meet the needs of the patients in the Morristown Memorial Facility”.* P.10. Exclusion criteria included brainstem CVA’s, patients with poor secretion management, patients with severe cognitive impairments. | - **Significant adaptions to the extent that it counters the original intention of the FWP** to allow free access to water to patients who aspirate but tolerate aspiration because of other risk mitigation strategies.” P.118 Murray et al 2022b.  **“***SLPs appeared to design the FWP to be as safe as possible e.g. tsps. of water and if there was no evidence of aspiration in instrumental assessment.”* - Kenedi et al. (2019) **“***The FWP guidelines were* ***modeled*** *after the protocol implemented at Frazier Rehabilitation Centre” (p.e11).* Kenedi adapted the design by offering **ice chips** or water to the experimental group. |
| 1. **Trialability – The degree to which the innovation can be tested or piloted on a small scale and undone**   Updated CFIR: Interview Guide Questions & Coding Guidelines (June 2023) - This construct captures the inherent trialability of the innovation not the need to trial nor the process of trialing the innovation. **Include statements about:** • The (in)ability to trial the innovation, due to features of the innovation itself. **Exclude statements about:** • Trialing the innovation, and instead code to Implementation Process: Doing. |  | - **Difficulty recruiting participants** (P.287) (Barker et al. 2019). **Exclusion criteria** has also been cited as a potential barrier to recruitment in Murray et al. (2022a). *“It is unclear whether the difficulty researchers had with recruitment to safety trials related to the acuity and complexity of patient presentations in care and their exclusion criteria or the nature of the acute setting itself rendering FWP implementation unfeasible"* P632.; Many participants working in the stroke units cited poor functional status in the areas of mobility, cognition or respiration as reasons not to implement the FWP. While this reasoning also aligns with the exclusion criteria of existing FWP studies, it effectively excludes a large proportion of patients in stroke and general medicine units in acute hospitals." SLPs - significant swallowing or oral issues may be considered a red flag and warrant exclusion - ""poor secretion management would be another one that I'd excluded, if they're needing lots of suctioning [S SLP5] P.637."" + level of alertness, impulsivity, delirium and fatigue were considered to potentially increase risk. - Nurses felt that patients on fluid restrictions would be unsuitable for a FWP. - Dietitians considered these patients suitable but changes may need to be made around the patient's non oral feeding, so the fluid restriction is not compromised. ""I don't envisage that people are going to have volumes - big volumes of water...It's not going to affect a fluid restriction you wouldn't imagine"" [S DN4] P.637. - **Short stay** - Patient are often discharged within a week from the acute care stroke unit P.10 Weber 2009. Kenedi (2019 e.16) identified short stay as a limitation to studies conducted in the acute care setting. |
| 1. **Complexity – The degree to which the innovation is complicated, which may be reflected by its scope and/or the nature and number of steps**   Updated CFIR: Interview Guide Questions & Coding Guidelines (June 2023) - I**nclude statements about:** · None specified **Exclude statements about**: • The complexity of implementation, and instead code to the relevant CFIR construct, e.g., if implementation is complex due to incompatibility with existing workflows, code to Compatibility. o Note: If the statement is about the complexity or difficulty of implementation influencing the likelihood of implementation success, code to Anticipated Implementation Outcomes: Implementability (see CFIR Outcomes Addendum). |  | - Complexity of patient selection and design of the FWP for that patient + translating a protocol which was designed for and mainly researched in one context (i.e. inpatient rehabilitation) into a different context (i.e. acute care), where there is little consensus regarding FWP implementation (P.118 Murray 2022b). |
| 1. **Design – The degree to which the innovation is well designed and packaged, including how it is assembled, bundled, and presented**   Updated CFIR: Interview Guide Questions & Coding Guidelines (June 2023) - **Include statements about**: • Quality of packaging, appearance, organization, and format of the innovation and associated physical, electronic, or online materials o • Types of materials bundled with the innovation including: o Branding/marketing materials, e.g., posters o Recipient materials, e.g., worksheets, user interfaces o Deliverer materials, e.g., pocket cards o Implementation materials, e.g., an implementation toolkit. Innovation materials may include information regarding: § Costs, resources, competencies, and training specifications are identified § Evidence supporting the effectiveness of the innovation § Recommended strategies to implement the innovation and engage deliverers and recipients § Instructions for deliverers, e.g., information is provided to help apply to individual recipients § Measures to evaluate the innovation **Exclude statements about**: • The presence or absence of supplies or materials, and instead code to Available Resources. Sharing, marketing, or branding innovation materials to engage individuals in implementation and/or delivery of the innovation, and instead code to Implementation Process: Engaging. • Actual provision of training, and instead code to Access to Knowledge and Information. | - **Instructions for deliverers** – The FWP specifies certain guidelines including regular oral care, which are designed to minimize the risk of adverse consequences. P. 632 (Murray 2022a).” Rules aimed at maximizing patient safety” P. 286 Barker 2019” - **Types of Materials bundled with the intervention** - (Kenedi et al. (2019) Administered the Aspiration Precaution Oral Care Program. Used in service and written material to educate nursing staff on the study and implementation's purpose; Weber (2009) *“A tracking sheet for oral care and water intake is kept at patient's bedside.”* |  |
| 1. **Cost – The degree to which the innovation purchase and operating costs are affordable**   Updated CFIR: Interview Guide Questions & Coding Guidelines (June 2023) - **Include statements about:** · Tangible costs to purchase, subscribe, or use the innovation; include cost of training associated with the innovation, e.g., registration. **Exclude statements about**: • Resources available to implement and deliver the innovation, and instead code to Available Resources. o Note: Innovation Cost should capture the expense of the innovation while Available Resources should capture whether the Inner Setting can or will provide resources to implement. Examples: • Costs related to conducting research (e.g., funding for research staff, participant incentives); these are outside the purview of the CFIR; however, a construct can be added to capture these themes. • Cost-effectiveness, and instead code to Innovation Evidence-Base or as an Innovation Outcome. | None mentioned | None mentioned |

**2. OUTER SETTING DOMAIN**

| **Construct Name and Construct Definition** | **Facilitator** | **Barrier** |
| --- | --- | --- |
| **A. Critical Incidents – The degree to which large-scale and/or unanticipated events disrupt implementation and/or delivery of the innovation.**  Updated CFIR: Interview Guide Questions & Coding Guidelines (June 2023) - **Include statements about**: · None specified **Exclude statements about**: · None specified | None mentioned | None mentioned |
| **B. Local Attitudes The degree to which Sociocultural values (e.g., shared responsibility in helping recipients) and beliefs (e.g., convictions about the worthiness of recipients) encourage the Outer Setting to support implementation and/or delivery of the innovation.**  Updated CFIR: Interview Guide Questions & Coding Guidelines (June 2023) - **Include statements about**: · Values and beliefs of the Outer Setting. o Sociocultural values include, e.g., individualism vs. collectivism, equitable vs. equal distribution of support. o Sociocultural beliefs include, e.g., biases, stigma, discrimination, and/or oppression resulting from e.g., racism, ableism. **Exclude statements about:** · None specified | None mentioned | None mentioned |
| **C. Local Conditions**  **Economic, environmental, political, and/or technological conditions enable the Outer Setting to support implementation and/or delivery of the innovation.**  Updated CFIR: Interview Guide Questions & Coding Guidelines (June 2023) - **Include statements about**: · None specified **Exclude statements about**: · None specified |  | - **Local Political Conditions** – Barker et al (2019) **Government enforced changes** to the state health system - ‘*Additional staffing requirements resulting from government enforced changes to the state health system (as well as* ***hospital admitting more patients*** *than the wards were staffed to manage has resulted in greater utilization of agency nurses who lack familiarity with the acute stroke environment’* (p291). |
| **D. Partnerships & Connections – The degree to which the Inner Setting is networked with external entities, including referral networks, academic affiliations, and professional organization networks.**  Updated CFIR: Interview Guide Questions & Coding Guidelines (June 2023) - **Include statements about:** · Information sharing and co-learning through formal or informal connections with individuals or groups outside the Inner Setting, including attending networking and/or education events, e.g., conferences and arrangements at the system-level **Exclude statements about:** · Relationships, networks, and teams inside the Inner Setting, and instead code to Relational Connections. · Communication and information sharing practices inside the Inner Setting, and instead code to Communications. | None mentioned | None mentioned |
| **E. Policies & Laws - The degree to which legislation, regulations, professional group guidelines and recommendations, or accreditation standards support implementation and/or delivery of the innovation.**  Updated CFIR: Interview Guide Questions & Coding Guidelines (June 2023) - **Include statements about**: · None specified **Exclude statements about**: · None specified | - **Professional guidelines and recommendations *-*** *National Institute for Health Care Excellence (NICE) positioned the avoidance of aspiration pneumonia as its top research priority for stroke, specifically recommending investigation into the benefits of FWPs versus nil by mouth or the use of thickened fluids (NICE, 2019).* P.632 Murray et al. (2022a) |  |
| **F. Financing – The degree to which**  **funding from external entities (e.g., grants, reimbursement) is available to implement and/or deliver the innovation.**  Updated CFIR: Interview Guide Questions & Coding Guidelines (June 2023) - **Include statements about:** · The availability of funding from the Outer Setting. **Exclude statements about:** · The availability of funding in the Inner Setting, and instead code to Available Resources: Funding. o Note: If the level of external funding influences the availability of funding in the Inner Setting, code both Financing and Available Resources: Funding. | None mentioned | None mentioned |
| **G. External Pressure - The degree to which external pressures drive implementation and/or delivery of the innovation. Note: Use this construct to capture themes related to External Pressures that are not included in the subconstructs below.**  Updated CFIR: Interview Guide Questions & Coding Guidelines (June 2023) - This construct can be used to elicit general information about External Pressures. More detailed information can be elicited or coded using the subconstructs below or by adding new subconstructs   1. Societal Pressure - Mass media campaigns, advocacy groups, or social movements or protests drive implementation and/or delivery of the innovation.   **Include statements about**: · None specified **Exclude statements about**: · None specified   1. Market Pressure - Competing with and/or imitating peer entities drives implementation and/or delivery of the innovation.   **Include statements about**: · Acting to “keep up with the Joneses”, i.e., to close a gap compared to another competitor or influential entity (whether gap is actual or perceived) **Exclude statements about:** · None specified   1. Performance-Measurement Pressure - Quality or benchmarking metrics or established service goals drive implementation and/or delivery of the innovation.   **Include statements about**: · Performance measures originating from the Outer Setting **Exclude statements about:** | None mentioned | None mentioned |

**3. INNER SETTING DOMAIN**

| Construct Name and Construct Definition | Facilitator | Barrier |
| --- | --- | --- |
| Constructs A – D exist in the Inner Setting regardless of implementation and/or delivery of the innovation, i.e., they are persistent general characteristics of the Inner Setting. |  |  |
| 1. Structural Characteristics - The degree to which Infrastructure components support functional performance of the Inner Setting. Note: Use this construct to capture themes related to Structural Characteristics that are not included in the subconstructs below. 2. Physical Infrastructure - Layout and configuration of space and other tangible material features support functional performance of the Inner Setting.   Updated CFIR: Interview Guide Questions & Coding Guidelines (June 2023) - Include statements about: · None specified Exclude statements about: · Availability of space, and instead code Available Resources: Space.   1. Information Technology Infrastructure - Technological systems for tele-communication, electronic documentation, and data storage, management, reporting, and analysis support functional performance of the Inner Setting.   Updated CFIR: Interview Guide Questions & Coding Guidelines (June 2023) - Include statements about: · Electronic Health Record (EHR) Systems, data warehouses, and data visualization tools. Exclude statements about: · Availability of IT equipment, and instead code Available Resources: Materials & Equipment   1. Work Infrastructure - Organization of tasks and responsibilities within and between individuals and teams, and general staffing levels, support functional performance of the Inner Setting.   Updated CFIR: Interview Guide Questions & Coding Guidelines (June 2023) - Include statements about: · Bureaucracy, reporting structure (power structures), delegation of tasks, schedules/shifts, order of tasks, workload, and work tempo (rate, rhythm or pattern of activities, pace). · General staffing levels in the Inner Setting, e.g., high staff turnover, understaffing. Exclude statements about: · Availability of time (e.g., dedicated time) to implement and/or deliver the innovation, and instead code Individuals: Role * Opportunity. o Note: If the general staffing levels in the Inner Setting influences the availability of time to implement and/or deliver the innovation, code both Structural Characteristics: Work Infrastructure and Individuals: Role * Opportunity. | - **3. Work Infrastructure - Work tempo (rate, rhythm or pattern of activities, pace):** **Pattern of activities** - Frequent contact between nursing and patients and regular patient monitoring. Barker et al. (2019). | - **3. Work Infrastructure - Work Tempo (rate, rhythm or pattern of activities, pace): "***Acute care is time intensive which makes it challenging for SLPS to educate clinicians" P.291* Barker et al. (2019). - **Workload:** The **additional workload** on an **existing heavy workload** required to implement WPs consistently reported as a barrier to implementation. + **heavy workload** impacting on oral care *- “Participants from the current study almost unanimously perceived WPs would result in an increased burden on nursing workload. They considered that scheduling oral care requirements, offering of water and supervising consumption into* ***an existing heavy workload*** *would be challenging, and could ultimately lead to a lack of adherence to the guidelines or may even result in WPs not being implemented at all” P292, " My concern would be more about when do you actually fit it in, that you can go on with the* ***workload that you’ve already got to do****. That’s why I feel like some nurses just wouldn’t do it [N4A] P291*; - **General staffing levels (e.g. high staff turnover, understaffing): Transient workforce** impacting on requirement for ongoing education for rotated staff and agency nurses. |
| 1. Relational Connections – The degree to which There are high quality formal and informal relationships, networks, and teams within and across Inner Setting boundaries (e.g., structural, professional).   Updated CFIR: Interview Guide Questions & Coding Guidelines (June 2023) - Include statements about: · Boundary spanning, teamness, and cohesion in the Inner Setting. · Social network analyses in the Inner Setting. Exclude statements about: · Relationships with individuals and/or entities in the Outer Setting, and instead code Partnerships & Connections. · Forming an implementation team, and instead code to Implementation Process: Teaming. | - **Teamness and cohesion -** *"Generally, I feel we do reasonably well here cause obviously* ***we are the stroke ward****. I guess that can sometimes sort of decrease a bit with relievers or new staff members that aren’t accustomed to the word"* N3. P116 Murray 2022b |  |
| 1. Communications – The degree to which there are high quality formal and informal information sharing practices within and across Inner Setting boundaries (e.g., structural, professional).   Updated CFIR: Interview Guide Questions & Coding Guidelines (June 2023) - Include statements about: · None specified Exclude statements about: · Receiving or not receiving information needed to implement and/or deliver the innovation, and instead code to Access to Knowledge & Information. o Note: If Access to Knowledge & Information is being influenced by existing communication structures and norms, code both Communications and Access to Knowledge & Information. · Communicating to engage individuals in implementation and/or delivery of the innovation, and instead code to Implementation Process: Engaging. | - **Established systems** to educate nursing staff about changes to current practice; processes to disseminate new protocols Barker et al. (2010) |  |
| 1. Culture – The degree to which there are shared values, beliefs, and norms across the Inner Setting. Note: Use this construct to capture themes related to Culture that are not included in the subconstructs below. 2. Human Equality-Centered - There are shared values, beliefs, and norms about the inherent equal worth and value of all human beings.   Updated CFIR: Interview Guide Questions & Coding Guidelines (June 2023) - Include statements about: · Diversity, equity, inclusion and belonging (or lack thereof) in the Inner Setting related to those who have been minoritized, marginalized, and/or oppressed based racism, sexism, heterosexism, cissexism, classism, ableism, sizeism and other biases. · Shared power and decision making (or lack thereof) in the Inner Setting. Exclude statements about: · None specified   1. Recipient-Centeredness - There are shared values, beliefs, and norms around caring, supporting, and addressing the needs and welfare of recipients.   Updated CFIR: Interview Guide Questions & Coding Guidelines (June 2023) - Include statements about: · The extent to which Recipient wishes, needs, and preferences drive implementation and/or adaptations to the innovation. o Note: If recipient wishes, needs, and preferences drive adaptations, code both Recipient-Centered Culture and Adapting. Exclude statements about: · Assessing Needs of Recipients as part of the implementation process, and instead code to Process: Assessing Needs.   1. Deliverer-Centeredness - There are shared values, beliefs, and norms around caring, supporting, and addressing the needs and welfare of deliverers.   Updated CFIR: Interview Guide Questions & Coding Guidelines (June 2023) - Include statements about: · The extent to which Deliverer wishes, needs, and preferences inform implementation and/or adaptations to the innovation. o Note: If deliverer wishes, needs, and preferences drive adaptations, code both Deliverer-Centered Culture and Adapting. · The extent to which Innovation Deliverers feel supported and valued in Inner Setting. Exclude statements about: Assessing Needs of Deliverers as part of the implementation process, and instead code to Process: Assessing Needs.   1. Learning-Centeredness - There are shared values, beliefs, and norms around psychological safety, continual improvement, and using data to inform practice.   Updated CFIR: Interview Guide Questions & Coding Guidelines (June 2023) - Include statements about: · The nature and quality of close and enduring exchanges between more experienced individuals with less experienced individuals to encourage learning and skills in improving processes and systems. · The extent to which the Inner Setting is data driven, i.e., learning health systems turn data into knowledge, knowledge into performance, performance to data. Exclude statements about: · Collecting and discussing quantitative and qualitive information about the success/effectiveness of implementation or the innovation, and instead code Reflecting & Evaluating. o Note: If implementation and/or innovation specific Reflecting and Evaluating is being influenced by a learning-centered culture in the Inner Setting, code both Culture: Learning-Centeredness and Reflecting & Evaluating. | - **4. Learning-Centeredness: Learning culture** on an acute stroke ward, whereby staff have expectations that they will learn new processes and procedures *"We're always learning something new and implementing new activities on the ward."* [N3A] P.290. Barker et al. 2019; “*The culture of the workplace also influenced clinical decisions about initiating an FWP particularly exposure to the practice of other clinicians and modelling from supervisors and peers”* P. 640, Murray 2022a. | - **3. Deliverer centeredness** - *“Participant’s own attitude to risk, and their experiences with previous patients, may further influence their decision-making. Minimising the risk of aspiration dominated the discourse of many of our participants, despite the widely accepted notion that not all aspiration leads to aspiration pneumonia (Langmore et al., 1998)”* P.642 Murray 2022a; Culture of routinely providing thickened fluids preventing clinicians considering alternative approaches that may result in better patient care.; **Beliefs:** Majority of participants expressed beliefs and attitudes about aspiration that were highly risk averse. Barker et al. 2019; *“As a general health service, we’re risk averse. And the logic of water and the FWP and the conditions you recommend water go against some of those built-in risk averse concerns we have SSLP3* P. 639 **-** but then describes practitioners with more experience being more confident P.639 Murray 2022a - **Knowing the self** - how health professionals' experience, confidence and understanding of their own limitations influenced their decision to recommend or implement a FWP. Fewer years of experience were associated with more caution and risk avoidance. Murray 2022a. |
| Constructs E – K are specific to the implementation and/or delivery of the innovation. |  |  |
| 1. Tension for Change – The degree to which the current situation is intolerable and needs to change.   Updated CFIR: Interview Guide Questions & Coding Guidelines (June 2023) - Include statements about: · Needing or not needing to implement the innovation. o Note: Tension for Change is often associated with other constructs, e.g., Innovation Recipient * Needs and/or External Pressures commonly drive Tension for Change. Exclude statements about: · The innovation being better or worse than existing programs, and instead code to Relative Advantage. | None mentioned | None mentioned |
| 1. Compatibility – The degree to which innovation fits with workflows, systems, and processes.   Updated CFIR: Interview Guide Questions & Coding Guidelines (June 2023) - Include statements about: · The level of fit between existing work processes and infrastructure and the innovation. o Note: If a lack of compatibility with Inner Setting workflows, systems, and processes drives adaptation, code both Compatibility and Adapting. Exclude statements about: · None specified | - *""Just basic nursing care" [give them a drink...sit them up...swab their mouths]. [N8C]* *P.291* Barker et al. 2019 | - **Work infrastructure – Work tempo (pace)** - The most common barrier identified was the fast pace, high turnover of patients and overall caseload impacting on nursing availability to follow through all recommendations for safe FWP implementation. *""That's going to be potentially another thing for nurses to have to do...they might be like, oh, well, it's just too hard, we're not going to give you your water or something. So if we're adding extra things for the nursing staff to do, I mean that could hinder the successfulness of the water protocol as well." [S DN3] P.640* Murray et al. (2022a); Staff maybe too busy to follow through with providing oral care (Weber, 2009).; **Delegation of tasks -** "*A major barrier to implementation was relying on other staff"* re dependence on feeding P.115 Murray (2022b).; **Understaffing** - “*Documenting water intake over the course of the study would have been ideal but limited resources precluded this” e13 Kenedi et al. 2019.* |
| 1. Relative Priority – The degree to which Implementing and delivering the innovation is important compared to other initiatives.   Updated CFIR: Interview Guide Questions & Coding Guidelines (June 2023) - Include statements about: · Having to juggle priorities in [Inner Setting] due to other responsibilities, including other initiatives happening at the same time.... Exclude statements about: · The innovation being/not being a priority for recipients compared to other issues, and instead code Innovation Recipients * Need. |  | - **Having to juggle priorities due to other responsibilities** *– “Sometimes that gets put down lower on the priority list.” (SLP2), P.115. SLPs perceived nurses to prioritize their other duties over FWP implementation. P.118* Murray (2022b). |
| 1. Incentive systems – The degree to which Tangible and/or intangible incentives and rewards and/or disincentives and punishments support implementation and delivery of the innovation.   Updated CFIR: Interview Guide Questions & Coding Guidelines (June 2023) - Include statements about: · Performance reviews, evaluations, appraisals, or mandates to help ensure accountability and/or tangible or intangible rewards or punishments. · Tangible extrinsic rewards/punishments: Promotions/demotions, pay raise/pay decrease.    · Intangible extrinsic rewards/punishments: Praise/criticism, recognition/lack of recognition. Exclude statements about: · Intrinsic incentives, and instead code to Individuals: Roles * Motivation. | None mentioned | None mentioned |
| 1. Mission Alignment – The degree to which Implementing and delivering the innovation is in line with the overarching commitment, purpose, or goals in the Inner Setting.   Updated CFIR: Interview Guide Questions & Coding Guidelines (June 2023) - Include statements about: · Degree of alignment between the innovation and the overarching commitment, purpose, or goals in the Inner Setting. Exclude statements about: · Degree of alignment between the innovation and performance measures originating from the Outer Setting, and instead code to External Pressure: Performance-Measurement Pressure. o Note: If Inner Setting individuals (e.g., High- or Mid-Level Leaders, Innovation Deliverers) embrace and “own” external performance measures in a way that is internalized within the Inner Setting, code both External Pressure: Performance-Measurement Pressure and Mission Alignment. | None mentioned | None mentioned |
| J. Available Resources – The degree to which resources are available to implement and deliver the innovation. Note: Use this construct to capture themes related to Available Resources that are not included in the subconstructs below.   1. Funding - Funding is available to implement and deliver the innovation.   Updated CFIR: Interview Guide Questions & Coding Guidelines (June 2023) - Include statements about: · The availability and accessibility of funding to implement and/ or deliver the innovation in the Inner Setting. Exclude statements about: · The availability of funding from sources in Outer Setting, and instead code Financing. o Note: If the level of external funding directly influences the availability of funding in the Inner Setting, code both Financing and Available Resources: Funding. · Availability of funding to conduct research (e.g., obtaining regulatory approvals); this is outside the purview of the CFIR; however, a construct can be added to capture these themes.   1. Space - Physical space is available to implement and deliver the innovation.   Updated CFIR: Interview Guide Questions & Coding Guidelines (June 2023) - Include statements about: · The availability and accessibility, including the configuration, location, and quality of space needed to implement and/or deliver the innovation. Exclude statements about: · The general layout and configuration of the Inner Setting, and instead code to Structural Characteristics: Physical Infrastructure. · The availability of space to conduct research (e.g., sufficiently private space to consent research participants); this is outside the purview of the CFIR; however, a construct can be added to capture these themes.   1. Materials and equipment - Supplies are available to implement and deliver the innovation.   Updated CFIR: Interview Guide Questions & Coding Guidelines (June 2023) - Include statements about: · The availability and accessibility of materials and equipment needed to implement and/or deliver the innovation. Exclude statements about: · The design and quality of innovation materials, and instead code to Innovation Design.    · Availability of materials and equipment to conduct research (e.g., use of monitoring devices specifically for research purposes); this is outside the purview of the CFIR; however, a construct can be added to capture these themes. | None mentioned | None mentioned |
| K. Access to Knowledge & Information – The degree to which Guidance and/or training is accessible to implement and deliver the innovation.  Updated CFIR: Interview Guide Questions & Coding Guidelines (June 2023) - Include statements about: · The availability, convenience, accessibility, and usefulness of training (e.g., online learning modules, live training, workshops, on-demand) and guidance (e.g., on-going real-time help, access to experts, informational materials, FAQs). Exclude statements about: · Existing communication structures and norms unrelated to implementation and/or delivery, and instead code to Communications. o Note: If Access to Knowledge & Information is being influenced by existing communication structures and norms, code both Communications and Access to Knowledge & Information. · Sharing information to engage individuals in implementation and/or delivery of the innovation, and instead code to Implementation Process: Engaging. | - **Education and training -** To ensure adherence to FWP protocol guidelines, educational in-house services provided by SLP to educate nursing staff and physicians on the study's purpose before initiation. Attendance was documented. Education provided to families and caregivers. Method of education included: **skilled instructional education, demonstration and handout and teach back**. Kenedi et al. 2019**;** SLP r**eviews the protocol** with the patient and family and nursing staff and nursing assistants. **Written material is provided** and nurses are required to take a **simple competency after being educated**. Weber 2009 - **Peer support and modelling** as an enabler to implementation. P.292 Barker et al. 2019 modelling from supervisors and peers. *""If you've worked with people who've done it before and you've seen it implemented well…you're going to do it straight up…if you've worked for 10 years and never seen anyone do it, then you're not so likely to do it. So, I think it's much more about cultures than years of experience* (Note non stroke statement). P640 Murray 2022a - **Clinical supervision** “I talked about with my **supervisor** and she often prompted me...Having someone that was more experienced who could suggest when it would be appropriate, absolutely was helpful in confidence” SLP6B P.291. Barker 2019. | - **Education - Difficult to educate and train all shifts** of nursing and assistants that may be involved with a particular patient on a given day as this changes daily Weber 2009; **time and logistics for ongoing staff** education perceived as a significant barrier to the implementation P. 291 Barker 2019; - **Not having a clear written protocol** - *"And in order to do it, sometimes I mean it can be a challenge cause obviously it's there but you don't have it fully charted somewhere that you have to mark it off. So I guess that sometimes makes it difficult and stuff when we probably sometimes do forget"* [N3]. P.116 Murray 2022b |

**4. INDIVIDUALS DOMAIN**

| **Construct Name and Construct Definition** | **Facilitator** | **Barrier** |
| --- | --- | --- |
| **Roles Subdomain** |  |  |
| 1. High-Level Leaders - Individuals with a high level of authority, including key decision-makers, executive leaders, or directors.   Updated CFIR: Interview Guide Questions & Coding Guidelines (June 2023) **- Include statements about:**  • Formal high-level leaders, i.e., individuals that are officially recognized and have a high-level of power based on hierarchy.  **Exclude statements about:**  • Formal mid-level leaders, i.e., individuals that are officially recognized and have a mid-level of power based on hierarchy, and instead code to Mid-Level Leaders.  • Informal leaders, i.e., individuals that are not officially recognized but have influence based on their reputation, and instead code to Opinion Leaders.  • Implementation leaders, i.e., individuals leading implementation of the innovation, and instead code to Implementation Leads.  o Note: If an Implementation Lead is also another kind of leader, code both roles. For example, if the individual leading implementation is also the head of a department in the Inner setting, code both Implementation Lead and Mid-Level Leader. |  |  |
| 1. Mid-Level Leaders - Individuals with a moderate level of authority, including leaders supervised by a high-level leader and who supervise others.   Updated CFIR: Interview Guide Questions & Coding Guidelines (June 2023) **- Include statements about:**  • Formal mid-level leaders, i.e., individuals that are officially recognized and have a mid-level of power based on hierarchy, and instead code to Mid-Level Leaders.  **Exclude statements about:**  • Formal high-level leaders, i.e., individuals that are officially recognized and have a high-level of power based on hierarchy.  • Informal leaders, i.e., individuals that are not officially recognized but have influence based on their reputation, and instead code to Opinion Leaders.  • Implementation leaders, i.e., individuals leading implementation of the innovation, and instead code to Implementation Leads.  o Note: If an Implementation Lead is also another kind of leader, code both roles. For example, if the individual leading implementation is also the head of a department in the Inner setting, code both Implementation Lead and Mid-Level Leader. | Senior Clinicians - Leadership by **senior clinicians** appear critical for change in practice. Murray 2022b. Leadership by senior clinicians in implementing FWPs in acute care settings may facilitate more widespread acceptance of FWPs in this [acute setting]. Murray 2022a. |  |
| 1. Opinion Leaders - Individuals with informal influence on the attitudes and behaviors of others.   Updated CFIR: Interview Guide Questions & Coding Guidelines (June 2023) **- Include statements about:**  • Informal leaders, i.e., individuals that are not officially recognized but have influence based on their reputation, and instead code to Opinion Leaders.  **Exclude statements about:**  • Formal high-level leaders, i.e., individuals that are officially recognized and have a high-level of power based on hierarchy.  • Formal mid-level leaders, i.e., individuals that are officially recognized and have a mid-level of power based on hierarchy, and instead code to Mid-Level Leaders.  • Implementation leaders, i.e., individuals leading implementation of the innovation, and instead code to Implementation Leads.  o Note: If an Implementation Lead is also another kind of leader, code both roles. For example, if the individual leading implementation is also the head of a department in the Inner setting, code both Implementation Lead and Mid-Level Leader. |  |  |
| 1. Implementation Facilitators - Individuals with subject matter expertise who assist, coach, or support implementation.   Updated CFIR: Interview Guide Questions & Coding Guidelines (June 2023) **- Include statements about:**  • None specified  **Exclude statements about:**  • None specified |  |  |
| 1. Implementational Leads - Individuals who lead efforts to implement the innovation.   Updated CFIR: Interview Guide Questions & Coding Guidelines (June 2023) **- Include statements about**:  • Implementation leaders, i.e., individuals leading implementation of the innovation, and instead code to Implementation Leads.  Note: If an Implementation Lead is also another kind of leader, code both roles. For example, if the individual leading implementation is also the head of a department in the Inner setting, code both Implementation Lead and Mid-Level Leader.    **Exclude statements about:**  • Formal high-level leaders, i.e., individuals that are officially recognized and have a high-level of power based on hierarchy.  • Formal mid-level leaders, i.e., individuals that are officially recognized and have a mid-level of power based on hierarchy, and instead code to Mid-Level Leaders.  • Informal leaders, i.e., individuals that are not officially recognized but have influence based on their reputation, and instead code to Opinion Leaders. | Use of dedicated **implementation leaders and champions** to facilitate uptake of evidence-based practice Barker 2019. | - **SLPs** - Mindset of SLPs as gatekeepers/WPs as sole domain of SLPs "when it actually relies on a whole gamut of staff" [SLP3B], this mindset may result in WP not being implemented for patients " Barker 2019. |
| 1. Implementation Team Members - Individuals who collaborate with and support the Implementation Leads to implement the innovation, ideally including Innovation Deliverers and Recipients.   Updated CFIR: Interview Guide Questions & Coding Guidelines (June 2023) **- Include statements about:**  • None specified  **Exclude statements about:**  • None specified | All health professionals were described as having a role in recommending and implementing and monitoring FWPs. Participants indicated that judgements about factors relating to decision making were often shaped by team support and availability of supervision. Murray 2022a |  |
| 1. Other Implementation Support - Individuals who support the Implementation Leads and/or Implementation Team Members to implement the innovation.   Updated CFIR: Interview Guide Questions & Coding Guidelines (June 2023) **- Include statements about:**  • None specified  **Exclude statements about:**  • None specified | **Family support** in assisting with positioning and oral hygiene and monitoring outcomes influences some participants in their decision to implement a FWP. *“Because some of the patients might not take it from a nursing staff. They might have a success with a family member, so definitely.”* SN6. P.638. Murray 2022a. | - **Family** *- “If you don’t feel like you can trust the family...to do the right things, you wouldn’t put them on a free water protocol”* S SLP8, P.638 Murray 2022a. - **Dieticians** considered they played a minimal role in comparison with other disciplines. *“Apart from the supplement I don’t think we’d have a direct impact on how it’s implemented at all”* P.289 Barker 2019. |
| 1. Innovation Deliverers - Individuals who are directly or indirectly delivering the innovation   Updated CFIR: Interview Guide Questions & Coding Guidelines (June 2023) **- Include statements about:**  • None specified  **Exclude statements about:**  • None specified |  | - **Nurses** - "*So whether they would see this [FWP] as their responsibility - they might see it as a speechie [SLP] thing and think, not our [nursing] problem" [DN2A].* P.289 Barker 2019. |
| I Innovation Recipients - Individuals who are directly or indirectly receiving the innovation.  Updated CFIR: Interview Guide Questions & Coding Guidelines (June 2023) **- Include statements about:**  • None specified  **Exclude statements about:**  • None specified |  |  |
| **Characteristics Subdomain** |  |  |
| 1. Need – The degree to which the individual(s) has deficits related to survival, well-being, or personal fulfillment, which will be addressed by implementation and/or delivery of the innovation   Updated CFIR: Interview Guide Questions & Coding Guidelines (June 2023) **- Include statements about:**  • None specified  **Exclude statements about:**  • None specified | - **Wellbeing**: Perceived benefits to patients QOL through comfort, normalization and preferences for care: *""I think it's important that we allow people to have the most normal life that they can have and if a little bit of water make life more normal, then I think from that perspective it's quite an important thing...if we're thinking about it purely just from a hydration point of view I don't think it's as crucial""* [DN4B] P.290; I*t’s [water] providing comfort, to aid in thinks like general comfort and wellbeing, but also hydration, mouthcare and quality of mouth, oral mucosa” SLP7C P. 290* Barker et al. 2019 improved mood, faster rehabilitation of swallowing impairment and increased hydration, improved oral hygiene. Hydration to a lesser degree due to the small sips of water provided by a WP. Barker 2019; Patient 3 "*"I gotta have the water…I'll dry up if I don't get the water""*. Murray 2022b; In patients in the terminal stage of their illness, participants changed focus to prioritize patient comfort ahead of safety (S MO4). Considering patient's **preferences for care as well as QOL.** *The FWP was described as an option to* provide **pts with choice** in their care which is often limited in the acute setting *'If we could try to decrease the amounts of limits that we put on patient's diets to a level that's as safe as possible, that would be ideal;'* S DN3 P. 637 Murray 2022a | - **Wellbeing:** Perceived negative patient outcomes - aspiration and development of chest complications, but not all participants were concerned with aspiratio**n "if the aspiration isn't developing into anything."** *[DN4B] P.290;* reduced cognition and impulsivity, further increasing the patient's risk. Barker 2019; **Patients preferences** were almost always outweighed by factors more aligned with the patient's medical condition and safety P. 642 Murray 2022a. |
| 1. Capability – The degree to which the individual(s) has interpersonal competence, knowledge, and skills to fulfill Role.   Updated CFIR: Interview Guide Questions & Coding Guidelines (June 2023) **- Include statements about:**  **•** Psychological and physical ability to fulfill role, e.g., knowledge, skills, decision-making.  **Exclude statements about:**  **•** Characteristics that are conferred onto individuals by the Inner or Outer Setting, e.g., the time and authority granted to the Implementation Lead to fulfill their role, and instead code to Opportunity. | - **IMPLEMENTATION TEAM - Competence**: Acute stroke ward, staffed by clinicians with high degree of **dysphagia expertise** perceived as an implementation enabler P.292; **Knowledge**: Knowledge of the FWP intervention was perceived as an enabler for delivering the intervention with half of participants reporting an awareness of WPs. Barker 2019; **Knowing the case** - Medical officer reported **confidence** **i**n trialing FWPs with all stroke patients if the patient was comfortable and not experiencing episodes of choking or excessive coughing "*"Well again, even if they do have a pneumonia or some other condition, they're swallowing their own saliva all the time, so I don't see it theoretically as being any different [S MO1].""* P.635 - NB this was not a common opinion of participants. Murray 2022a. - **IMPLEMENTATION LEADS** - SLPs: **Prior experience of the FWP made it quicker and easier to implement "***I've been using it for a while that I'm quite confident and quite experienced enough to run with it...comes quite easily about my decision making. I think if you've got someone like a new grad who's not experienced with it then it might be a bit daunting." [SLP2A] P.289* Barker 2019; **Experience** led them to practice more or less conservatively with respect to acceptance of risk, including the FWP - greater experience was associated with increased confidence in the ability to determine suitable patients and implement the FWP in the acute setting P.639 - *'From a personal perspective I would l want - I'd be happy to aspirate thin fluids... only because I know the literature so well.'* P.639 Murray 2022a - **INNOVATION DELIVERER** – **Skills** - Nurses skills developed working with the acute stroke population would contribute positively to implementation i.e. stroke specific skills; *""In my expertise, because I have been doing [nursing] for so long, I would find it easy to implement and do it no problem. Quite confident to [implement WPs] and educating someone. Because I know the signs of deterioration, what to look for in aspiration." [N6B]* P.289 Barker et al. 2019 | - **IMPLEMENTATION TEAM - Lack of awareness of FWP** reported by 11 participants (from dietetics, medicine and nursing perceived as a barrier) - the sporadic and informal approach [thinks links with access to knowledge – see INNER SETTING – K] with which SLPS recommended WPs was a likely contributor to the lack of awareness by other professionals. - **IMPLEMENTATION LEADS** - Lack of clear documentation (i) a lack of clarity of instructions from SLP to nursing staff, which limited nursing staff ability to implement the FWP as intended. P.118 Murray 2022b. - **IMPLEMENTATION DELIVERERS** – Lack of clear documentation - incomplete or unclear documentation of how the FWP was implemented by nursing staff, which limited SLP ability to monitor the impact of the FWP on the client. P.118 Murray 2022b. - L**ack of understanding of stroke population needs re agency nurses.** "*"Management say, stroke isn't a specialty area. Well they need to come and work in our ward….that deficit of the throat is a huge risk if a new nurse or a student, or an agency nurse comes on, there can be difficulties in what they comprehend as giving them water"" [N5B] P.289*, concerns that WP recommendations be misinterpreted by other staff and family members; **Lack of experienced staff;** Nurses *"Most nurses working in acute stroke care lack the skills to complete oral care to the level required for compliance with a WP" P.289* Agency nurses: "most lack the stroke specific skills required to work with patients with acute stroke wards, increasing the likelihood of errors occurring if WPs were implemented.". Barker 2019; - **Nurses remembering to get the water** "Overlooked the offer of water altogether (P3)" P.118. Nurses not implementing the FWP as intended. - **Measures to mitigate aspiration risk sometimes not completed or documented** - this related mainly to oral care, close monitoring via chest auscultation, blood test results and/or fluid intake not being completed or documented. Murray 2022b. - **Knowing the case/Pattern of illness scripts** perpetually reinforcing the predominant practice pattern of not implementing the FWP in acute settings. *“Often with bleeds are sicker so they might be more drowsy and fluctuate more. People with deep thalamic strokes will fluctuate more [SN8] P.633” Murray 2022a.* |
| 1. Opportunity – The degree to which the individual(s) has availability, scope, and power to fulfill Role.   Updated CFIR: Interview Guide Questions & Coding Guidelines (June 2023) **- Include statements about:**  **•** Characteristics that are conferred onto individuals by the Inner or Outer Setting, e.g., the time and authority granted to the Implementation Lead to fulfill their role.  **Exclude statements about:**  **•** General staffing levels in the Inner Setting, e.g., high staff turnover, understaffing, and instead code Structural Characteristics: Work Infrastructure.  o Note: If the general staffing levels in the Inner Setting influences the availability of time to implement and/or deliver the innovation, code both Structural Characteristics: Work Infrastructure and Individuals: Role * Opportunity.  • Availability of time to conduct research; this is outside the purview of the CFIR; however, a construct can be added to capture these themes. | - **INNOVATION DELIVERS** - **Scope** (and confidence) to fulfill role "I'd be confident with that because it's - that's what I do." [DR2A]. "Most considered it an extension of their current role so felt they already had the required abilities." Barker 2019 - **OTHER IMPLEMENTATION SUPPORT –** availability of family support in assisting with positioning and oral care and monitoring outcomes**.** Barker 2019 | - **INNOVATION DELIVERERS – Availability** *- “But the bit I don’t see time for...is the mouth care and the rest of it. If they have to sit there and supervise sips of water, I can just see them [nursing] hand them a cup of water and walk out of the door...so steps are going to get missed* [DN2A] P. 290 Barker et al. 2019. Time to sit patients to sit up might preclude implementation *“Its the time pressure around that...with dense left sided weakness… for them to be able to sit upright that can often involve 4 individuals for one person to have a sip of water” SSLP4* P.637 Murray et al. (2022a); - Nursing availability to follow through all recommendations for safe FWP implementation, *""That's going to be potentially another thing for nurses to have to do...they might be like, oh, well, it's just too hard, we're not going to give you your water or something. So if we're adding extra things for the nursing staff to do, I mean that could hinder the successfulness of the water protocol as well." [S DN3] P.640* Murray et al. (2022a); Staff maybe too busy to follow through with providing oral care (Weber, 2009). |
| 1. Motivation - The degree to which the individual(s) is committed to fulfilling Role.   Updated CFIR: Interview Guide Questions & Coding Guidelines (June 2023) **- Include statements about:**  **•** Receptivity, desire, and/or dedication to fulfilling role.  **Exclude statements about:**  **•** None specified | - **INNOVATION DELIVERERS - Receptivity** - *"I think we're obliged to make time. It's our job, and all of the allied health team have got their job to do, so personally I think you've got to make time" [S N5]. P.640.* Murray 2022a. - **INNOVATION RECIPIENTS** - Patient **desire/receptivity** *""No, I gotta have the water…I'll dry up if I don't get the water."" [P3]*; patients prefer water; patients want to have access to water; water helps counteract thirst and dry mouth Murray 2022b, Personal values and perspectives regarding their own care influencing decisions to implement FWPs. Murray 2022a; Patients **knowing they would have access to water later may encourage adherence to thickened fluids** during mealtimes Murray 2022a; **Positive feedback** - Feedback from patients that have received water was positive and all felt their medical quality of life improved. Weber 2009. | - **INNOVATION DELIVERS - Concerns about legal liability and personal protection** (Nurses) if a WP negatively impacted on a patient's outcome. "Uncertainty regarding liability made these participants feel uncomfortable and less willing to implement WPs" *""this person's length of stay is increased. If we're doing it and their outcome isn't where the outcome might have gone had we not done it. So the legalities...it's that safety aspect for clinicians that's really important"" [N3A] P.290;* Barker 2019 *""If it all goes south because this person's length of stay is increased and if we're doing it [FWP] and their outcomes isn't where their outcome would have gone had we not done it"" [S N3]*P. 639 Murray 2022a; **Negative attitudes by staff about completing oral care** *"Nurses often view oral care as an unpleasant task and with reduced appreciation of the positive impacts to patient health status and wellbeing, oral care in the acute setting is often forgotten, neglected or viewed as a lower priority in comparison to other duties." P.292* Patients - refusing oral care. P.292 Barker 2019 ; **Motivation**: SLPs may be **reluctant** to refer a patient due to the amount of time it takes to set the patient up for the protocol i.e. getting MD orders, obtaining patient consent/training pt/family and health care providers/putting up tracking sheets in patients rooms/monitoring vitals on a daily basis, given nurses competencies. Weber, 2009. - **INNOVATION RECIPIENTS** - P3's misgivings about the discomfort and risk of pneumonia; water "gets stuck" and makes the patient cough; patients aware that aspiration may cause pneumonia Murray 2022b; |

**5. IMPLEMENTATION PROCESS DOMAIN**

| **Construct Name and Construct Definition** | **Facilitator** | **Barrier** |
| --- | --- | --- |
| 1. **Teaming – The degree to which individuals join, intentionally coordinating on interdependent tasks to implement the innovation.**   Updated CFIR: Interview Guide Questions & Coding Guidelines (June 2023) **- Include statements about:**  • Team dynamics, functioning, level of coordination and collaboration to implement the innovation.  **Exclude statements about:**  • Boundary spanning, social network analyses, teamness, and cohesion in the Inner Setting not related to the implementation process, and instead code to Relational Connections.  • The roles and characteristics of individual team members, and instead code to the appropriate constructs in the Individuals domain. | - Communication and **collaboration** among nurses, physicians, and SLPs will be necessary to monitor participant status across multiple floors and units. e17 Kenedi 2019. - All health professionals were described as having a role in recommending and implementing and monitoring FWPs. Participants indicated that judgements about factors relating to decision making were often shaped by **team support** and availability of supervision. Murray 2022a. | Mindset of SLPs as gatekeepers/WPs as sole domain of SLPs "when it actually relies on a whole gamut of staff" [SLP3B], this mindset may result in WP not being implemented for patients " Barker 2019.  "*So whether they would see this [FWP] as their responsibility - they might see it as a speechie [SLP] thing and think, not our [nursing] problem" [DN2A].* P.289 Barker 2019.  Dieticians considered they played a minimal role in comparison with other disciplines. *“Apart from the supplement I don’t think we’d have a direct impact on how it’s implemented at all”* P.289 Barker 2019. |
| 1. **Assessing Needs – The degree to which individuals collect information about priorities, preferences, and needs of people.** 2. Innovation Deliverers – The degree to which individuals collect information about the priorities, preferences, and needs of deliverers to guide implementation and delivery of the innovation.   Updated CFIR: Interview Guide Questions & Coding Guidelines (June 2023) **- Include statements about:**  **•** Informal or formal interviews or surveys with Innovation Deliverers.  **Exclude statements about:**  **•** The characteristics and/or priorities, preferences, and needs of Innovation Deliverers, and instead code to Individuals: Innovation Deliverers * Characteristics.   1. Innovation Recipients - The degree to which individuals collect information about the priorities, preferences, and needs of recipients to guide implementation and delivery of the innovation.   Updated CFIR: Interview Guide Questions & Coding Guidelines (June 2023) **-** **Include statements about**:  • Informal or formal interviews or surveys with Innovation Recipients.  **Exclude statements about:**  • The characteristics and/or priorities, preferences, and needs of Innovation Recipients, and instead code to Individuals: Innovation Recipients * Characteristics. | Barker et al. (2 SLP students) conducted semi structured interviews with 26 clinicians (Nurses, SLPs, doctors, dietitians) to identify clinicians' perceptions of the enablers of, and barriers to, successful implementation of WPs in acute stroke care.  Murray 2022a explored the explore the perceptions and decision-making process of clinicians about using FWPs to manage dysphagia for patients admitted to acute stroke and general medicine using semi structured interviews with dietitians, medical officers, registered nurses and SLPs.  Murray 2022b evaluated how SLPs arrive at decision making suitability relating to the FWP, how nursing staff implement the FWP, how SLPs and nursing staff experience this process (perceived barriers and enablers) and how patients experience the FWP using information from medical records and semi structured interviews with patient-nurse and SLP triads. | Weber methods for gathering recipient feedback about health related QOL unclear.  Kenedi did not collect information about priorities, preferences and needs of people. |
| 1. **Assessing Context – The degree to which individuals collect information to identify and appraise barriers and facilitators to implementation and delivery of the innovation.**   Updated CFIR: Interview Guide Questions & Coding Guidelines (June 2023) **-** **Include statements about:**  • Use of the CFIR to assess context 😉  **Exclude statements about:**  • None specified | Barker et al. analysed data was thematically and mapped themes deductively to the **Theoretical Domains Framework** (Cane et al., 2012).  Murray 2022a used the **Situated Clinical Decision Making Framework** (Gillespie & Peterson) to explore the complexity of clinical decision making and to identify barriers and facilitators.  Murray 2022b analysed the data descriptively and triangulated across sources. | Kenedi referred to the challenges of conducting a FWP research study in the acute setting as part of the study’s limitations.  Weber reflected on the challenges faced in the acute setting. |
| 1. Planning – The degree to which individuals identify roles and responsibilities, outline specific steps and milestones, and define goals and measures for implementation in advance. | None mentioned | None mentioned |
| 1. Tailoring Strategies – The degree to which individuals choose and operationalise implementation strategies to address barriers, leverage facilitators, and fit context.   Updated CFIR: Interview Guide Questions & Coding Guidelines (June 2023) **- Include statements about:**  • Making strategies operational for the Inner Setting. **Exclude statements about:** • None specified | - **Implementational Toolkit** – Tracking sheet for oral care and water kept at bedside for staff and or caregivers to fill out. Webber 2009. - **Communication and collaboration** among nurses, physicians, and SLPs to monitor participant status e.17 Kenedi 2019. - **Education** Providing the family with education to implement the strategies - “*her husband is generally always around so he is a good advocate for her and providing him with the education is a good avenue to allow it to be implemented more successfully”* SLP1 P.114 Murray 2022b; Individual education to participants, caregivers and nurses when necessitated by staff turnover. e11 Kenedi 2019. - **Implementation of thorough oral care protocols** before WPs are implemented in acute stroke. e12 Kenedi 2019. - **Monitor patients daily** for signs and symptoms of aspiration including i**ncreased temperatures, increased cough or congestion, positive chest x –rays and elevated white cell counts**. P.10 Weber 2009. |  |
| 1. **Engaging – The degree to which individuals attract and encourage participation in implementation /or the innovation.** 2. Innovation Deliverers – The degree to which individuals attract and encourage deliverers to serve on the implementation team and/or deliver the intervention.   Updated CFIR: Interview Guide Questions & Coding Guidelines (June 2023) - **Include statements about:**  • Strategies used to engage and retain Innovation Deliverers, e.g., sharing/disseminating information or evidence about the innovation, branding or marketing materials, and/ or “selling” the innovation.  **Exclude statements about:**  • The degree of delivery or enactment of expected behaviors by Innovation Deliverers, and instead code Actual Implementation Outcomes.   1. Innovation Recipients - The degree to which individuals attract and encourage recipients to serve on the implementation team and/or deliver the intervention.   Updated CFIR: Interview Guide Questions & Coding Guidelines (June 2023) - **Include statements about:**  • Strategies used to engage and retain Innovation Recipients, e.g., sharing information or evidence about the innovation, branding or marketing materials, and/ or “selling” the innovation.  **Exclude statements about:**  • The degree of receipt or enactment of expected behaviors by Innovation Recipients, and instead code to Innovation Outcomes: Recipient Impacts. | 2. **Innovation Recipients** – *“Letting the patient know as well so that they’re aware that’s something they can have. So that if they’re wanting that they can also kind of ask the nurses to provide that for them in case the nurses forget”* (SLP3). P.116 Murray 2022b. |  |
| 1. **Doing The degree to which individuals implement in small steps, test, or cycles of change to trial and cumulatively optimize delivery of the innovation.**   Updated CFIR: Interview Guide Questions & Coding Guidelines (June 2023) - This construct captures the process of trialing the innovation, not the need to trial nor the inherent trialability of the innovation.    **Include statements about:**  **•** None specified    **Exclude statements about:**  **•** The (in)ability to trial the innovation, due to features of the innovation itself, and instead code to Innovation Trialability. | Kenedi 2019 RCT – Implementation Design - Phase 1 Candidacy – Step 1 identify potential candidates by diagnosis; Step 2 – SLP assessment, Step 3 – exclusion criteria and additional considerations; Phase 2 Education; Phase 3: Implementation: Initiate Oral Care Programme. Initiate FWP with physician order. Track data. Monitor tolerance.  Weber 2009 Pilot study - All patients are evaluated by SLP to determine if meet the inclusion criteria. Discussion with the attending physician and an order for the protocol. Patient and family consent obtained, SLP reviews the protocol with patient and family and the nursing staff, including assistants. Written material is provided, and nurses are required to take a simple competency after being educated. To date at time of publication 5 patients recruited. Tracking sheet for oral care and water kept at bedside. Patients are followed daily and monitored for signs and symptoms of aspiration. |  |
| 1. **Reflective and Evaluating – The degree to which individuals collect and discuss quantitative and qualitative information about the success of the implementation.** 2. Implementation - The degree to which individuals collect and discuss quantitative and qualitative information about the success of the implementation.   Updated CFIR: Interview Guide Questions & Coding Guidelines (June 2023) **- Include statements about:**  • Time spent reviewing, discussing, and/or analyzing (i.e., making sense of) information related to the progress and/or success of implementation.  **Exclude statements about:**  • The actual progress and/or success of implementation, and instead code to Actual Implementation Outcomes (see CFIR Outcomes Addendum).  • The extent to which the Inner Setting has shared values around continual improvement and using data to inform practice, and instead code to Culture: Learning-Centeredness Culture.  o Note: If implementation and/or innovation specific Reflecting and Evaluating is being influenced by a learning-centered culture in the Inner Setting, code both Culture: Learning-Centeredness and Reflecting & Evaluating.  • Setting implementation goals, and instead code to Planning.  • An individual’s reflections as a part of data collection (e.g., participating in an interview), and instead code to the appropriate construct based on the content of the reflection.   1. 2. Innovation - The degree to which individuals collect and discuss quantitative and qualitative information about the success of the innovation.   Updated CFIR: Interview Guide Questions & Coding Guidelines (June 2023) **- Include statements about:**  • Time spent reviewing, discussing, and/or analyzing (i.e., making sense of) information related to the progress and/or success of the innovation.  **Exclude statements about:**  • The actual progress and/or success of the innovation, and instead code to Innovation Outcomes (see CFIR Outcomes Addendum).  • The extent to which the Inner Setting has shared values around continual improvement and using data to inform practice, and instead code to Culture: Learning-Centeredness Culture.  o Note: If implementation and/or innovation specific Reflecting and Evaluating is being influenced by a learning-centered culture in the Inner Setting, code both Culture: Learning-Centeredness and Reflecting & Evaluating.  • An individual’s reflections as a part of data collection (e.g., participating in an interview), and instead code to the appropriate construct based on the content of the reflection. | 1. **Implementation** - **Interdisciplinary approach** - Combining the use of champions with an interdisciplinary approach, where team members work together to develop patient care plans, detailing duties and accountability for oral care, education, supervision, safe swallowing strategies, documentation, water provision and individual patient fluid requirements, **may facilitate the implementation** of WPs in the acute stroke environment P.293 Barker 2019.   **Interdisciplinary negotiation** “where the roles of each discipline are clearly defined, and responsibilities are clearly enacted and documented will allow a better integration of patient care across disciplines and thus provide a more promising framework for the successful implementation of FWPs in the acute setting.” P.118. Murray 2022b; **Communication and education:** *“Maintaining regular communication between clinicians, in particular at handover, (and continuing efforts to educate rotating and permanent staff) will contribute to maximizing adherence for patients recommended a WP as part of their dysphagia management strategy” P. 292* Barker et al. 2019; *“considerable education on WP rules, risks and benefits would facilitate adherence to guidelines and minimise the likelihood of adverse patient outcomes.”* *P. 292* Barker et al. 2019; **Strategies to alleviate nursing workload** – Offer water in lieu of thickened fluids between meals; support staff and family members to offer and supervise patients who are consuming water in line with SLP recommendations P. 292 Barker 2019. **Implementation of thorough oral care protocols** before WPs are implemented in acute stroke P.292 Barker, 2019, P.118 Murray 2022b; **Formalised guidelines and protocols** for implementing FWPs with (whom, when and how) to allow uptake. P. 642 Murray 2022a  **Leadership** by **senior clinicians** Murray 2022a. **Modelling** of FWP decision making and implementation **by senior staff** to influence attitude to risk and encourage use P.642 Murray 2022a. **Patient outcomes and experiences** - **Address issues related to monitoring and supporting hydration** P.119 Murray 2022b - Involving patients in the decision making regarding FWPs P. 119. Murray 2022b.   - **Future research** into the perceptions of patients and their families about how they weigh up choice versus safety is needed. P.642 Murray 2022a. | 1. **Implementation** - **Possibility that the uncertainty as to whether FWP conditions would be implemented as recommended** e.g. oral care, close monitoring via chest auscultation, blood test results and/or fluid intake not being completed/documented contributed to the conservative or risk averse decision making by SLPs observed in this study (Murray 2022b). 2. **Innovation - Diagnosis based exclusion criteria** need for broader consideration of patient function rather than diagnosis based exclusion criteria alongside modifiable context specific factors (Murray 2022b).  - **Future research** will require adherence to oral care and free water protocols and education of multidisciplinary staff members. Communication and collaboration among nurses, physicians, and SLPs will be necessary to monitor participant status across multiple floors and units. E.17 Kenedi 2019. |
| I. **Adapting – The degree to which individuals modify the innovation and/or the Inner Setting for optimal fit and integration into work processes.**  Updated CFIR: Interview Guide Questions & Coding Guidelines (June 2023) **-** This construct captures the process of adapting the innovation and/or Inner Setting, not the need to adapt nor the inherent adaptability of the innovation. Users may wish to add new subconstructs based on a reporting framework for adaptations, e.g., the FRAME.    **Include statements about:**  **•** Adapting the Inner Setting, e.g., changing Inner Setting work processes or building infrastructure to accommodate the innovation, or innovation, e.g., changing innovation components or processes.  o Note: If adaptations are driven by other CFIR constructs, code both constructs. For example:   If recipient wishes, needs, and preferences drive adaptations, code both Recipient-Centered Culture and Adapting.   If deliverer wishes, needs, and preferences drive adaptations, code both Deliverer-Centered Culture and Adapting.   If a lack of compatibility with Inner Setting workflows, systems, and processes drives adaptation, code both Compatibility and Adapting.  **Exclude statements about:**  • The (in)ability to adapt the innovation, e.g., complaints about the rigidity of the protocol, due to features of the innovation itself or lack of “permission” to change components, and instead code to Innovation Adaptability.  • The innovation needing/not needing to be adapted, and instead code to the relevant CFIR construct, e.g., if an innovation needs/needed to be adapted because it did not meet recipients’ needs, code to Innovation Recipient: Needs; if an innovation needs/needed to be adapted because it did not fit with existing workflows, code to Compatibility.  • Adapting implementation strategies and/or implementation processes, and instead code to the relevant strategy or process being adapted, e.g., changes to the implementation plan would be coded to Planning. |  | Adaptations were made at multiple levels...this likely reflects the complexity and limitations of translating an intervention protocol designed for and mainly researched in one context (i.e. inpatient rehabilitation) into a different context (i.e. acute care), where there is little consensus regarding implementation. P.118 Murray et al. 2022b. |

**References**

Barker A, Doeltgen S, Lynch E, Murray J. Perceived barriers and enablers for implementing water protocols in acute stroke care: A qualitative study using the Theoretical Domains Framework. Int J Speech Lang Pathol. 2019 Jun;21(3):286-294. doi: 10.1080/17549507.2019.1595145. PMID: 31213158.

Kenedi H, Campbell-Vance J, Reynolds J, Foreman M, Dollaghan C, Graybeal D, Warren AM, Bennett M. Implementation and Analysis of a Free Water Protocol in Acute Trauma and Stroke Patients. Crit Care Nurse. 2019 Jun;39(3):e9-e17. doi: 10.4037/ccn2019238. PMID: 31154338.

Murray J, Maloney S, Underdown K, Doeltgen S. Patient suitability for free water protocols in acute stroke and general medicine: a qualitative study of clinician perceptions. Int J Lang Commun Disord. 2022a May;57(3):630-644. doi: 10.1111/1460-6984.12713. Epub 2022 Mar 23. PMID: 35318783.

Murray J, Walker C, Doeltgen S. Implementation of free water protocols in acute care: An observation of practice. Int J Speech Lang Pathol. 2022b Apr;24(2):111-121. doi: 10.1080/17549507.2021.1955973. Epub 2021 Aug 3. PMID: 34343448.

Weber V. The challenges of initiating the Frazier Water Protocol on an acute care stroke unit. Institute for Nursing Newsletter. 2009;5(3):10.
